# Cell‐Laden Multiple‐Step and Reversible 4D Hydrogel Actuators to Mimic Dynamic Tissue Morphogenesis

**DOI:** 10.1002/advs.202004616

**Published:** 2021-03-01

**Authors:** Aixiang Ding, Oju Jeon, Rui Tang, Yu Bin Lee, Sang Jin Lee, Eben Alsberg

**Affiliations:** ^1^ Department of Biomedical Engineering Case Western Reserve University 10900 Euclid Avenue Cleveland OH 44106 USA; ^2^ Department of Orthopaedic Surgery Case Western Reserve University 10900 Euclid Avenue Cleveland OH 44106 USA; ^3^ Present address: Richard and Loan Hill Department of Biomedical Engineering University of Illinois at Chicago 909 S. Wolcott Ave. Chicago IL 60612 USA; ^4^ Present address: Departments of Mechanical and Industrial Engineering, Orthopaedics, and Pharmacology University of Illinois at Chicago 909 S. Wolcott Ave. Chicago IL 60612 USA

**Keywords:** 4D biomaterials, biomimicry, controllable and programmable actuation, morphodynamic tissue engineering, morphogenesis

## Abstract

Shape‐morphing hydrogels bear promising prospects as soft actuators and for robotics. However, they are mostly restricted to applications in the abiotic domain due to the harsh physicochemical conditions typically necessary to induce shape morphing. Here, multilayer hydrogel actuator systems are developed using biocompatible and photocrosslinkable oxidized, methacrylated alginate and methacrylated gelatin that permit encapsulation and maintenance of living cells within the hydrogel actuators and implement programmed and controlled actuations with multiple shape changes. The hydrogel actuators encapsulating cells enable defined self‐folding and/or user‐regulated, on‐demand‐folding into specific 3D architectures under physiological conditions, with the capability to partially bioemulate complex developmental processes such as branching morphogenesis. The hydrogel actuator systems can be utilized as novel platforms for investigating the effect of programmed multiple‐step and reversible shape morphing on cellular behaviors in 3D extracellular matrix and the role of recapitulating developmental and healing morphogenic processes on promoting new complex tissue formation.

## Graphical abstract

Multilayered cell‐laden hydrogel actuators (CHAs) are developed to mimic dynamic tissue morphogenesis. The CHAs, which show multiple deformations programmably and controllably, enable defined self‐folding and/or user‐regulated, on‐demand‐folding into specific 3D architectures under physiological conditions. With these multilayered cytocompatible systems, potential applications in the biomedical field, such as morphodynamic tissue engineering, are demonstrated.

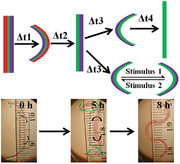

## 1 Introduction

Continuous changes in tissue and organ architecture, such as reorganization,^[^ ^1^ ^]^ remodeling,^[^ ^2^ ^]^ and morphogenesis,^[^ ^3^ ^]^ occur throughout development.^[^ ^4^ ^]^ The resulting shapes of tissues and relative positions of different cell types, extracellular matrix molecules, and soluble factors ultimately contribute to their functionality.^[^ ^5^ ^]^ For this reason, biomaterials and tissue engineering strategies that possess characteristics enabling morphological changes that biomimic these natural processes may enhance our ability to form complex tissues that structurally resemble native ones.^[^ ^6^ ^]^ However, the current state of tissue engineering, which holds great potential for replacement of damaged or lost tissues/organs,^[^ ^7^ ^]^ generally relies on geometrically static materials that are unable to recapitulate the aforementioned critical dynamic behaviors. Given the significance of the dynamic nature of tissues and organs during their development to achieve their mature structure, there has recently been great interest in incorporating dynamic shape changing capabilities into biocompatible materials.^[^ ^8^ ^]^ To make the materials intrinsically maneuverable and programmable, hydrogel actuators that can change their shape have been engineered for multiple applications in biomedical engineering.^[^ ^9^ ^]^ In contrast to geometrically static materials, hydrogel actuators respond to external stimuli by changing their shape. The capacity to temporally manipulate the actuator spatial structure and composition positioning may be valuable in guiding development‐inspired morphogenetic processes during organoid formation and engineering of tissues.

Typically, external stimuli, such as changes in light wavelength,^[^ ^10^, ^11^ ^]^ media pH,^[^ ^12^ ^]^ environmental temperature,^[^ ^13^ ^]^ exogenous chemicals,^[^ ^14^ ^]^ applied electricity,^[^ ^15^ ^]^ magnetic fields,^[^ ^16^ ^]^ and specific biochemical signals,^[^ ^17^ ^]^ modulate 4D hydrogel swelling and/or shrinking and produce macroscopic actuation. The integration of one or multiple stimuli into such responsive systems, such as gradient hydrogels,^[^ ^18^ ^]^ single‐component hydrogels (unimorph),^[^ ^19^ ^]^ and bilayer^[^ ^20^ ^]^ and trilayer^[^ ^21^ ^]^ hydrogels, can impel the soft actuators to explicitly go through shape evolvement over time either in a preprogrammable fashion, referring to a defined shape morphing in the presence of predefined environmental conditions, or in an “on‐demand” manner, referring to a regulated deformation under application of user‐controlled external stimulation. Taking advantage of a stimuli responsive systems’ programmability and controllability together with a specific geometrical design, hydrogel actuators are cast into 2D or 3D architectures^[^ ^22^ ^]^ to carry out specific functions (e.g., transporting a cargo,^[^ ^23^ ^]^ flow control,^[^ ^24^ ^]^ and grasping^[^ ^25^ ^]^) or locomotion (e.g., swimming^[^ ^26^ ^]^, walking^[^ ^27^ ^]^, and twisting^[^ ^28^ ^]^). However, no 4D system, noncytocompatible or cytocompatible, has been reported that is capable of multiple preprogrammable deformations. Furthermore, while 4D systems that undergo multiple “on‐demand” directional shape changes in response to external stimulation have been demonstrated, these systems were noncytocompatible or not demonstrated to be cytocompatible.^[^ ^29^, ^30^ ^]^

Although hydrogel actuators exhibit great potential in tissue engineering,^[^ ^31^ ^]^ drawbacks of the current systems heavily restrict their practical applications. For example, nonbiocompatible and/or cytotoxic materials and techniques are often used for hydrogel fabrication and/or harsh conditions, such as low pH, high temperature, and toxic chemicals and solvents, are needed to activate shape responses.^[^ ^32^, ^33^ ^]^ Hydrogel actuators designed for tissue engineering applications aimed at replicating aspects of tissue morphogenesis should meet important criteria such as: a) mechanical integrity that allows repeatable shape manipulation and high resistance to complex cell‐culture environments and long‐time incubation; b) cyto‐ and biocompatible stimulation that empowers their spatiotemporal tunability under biological conditions; c) a simple fabrication method that enables reproducible shape change response outcomes. Some cell‐laden hydrogel actuators (CHAs) fabricated by using biocompatible materials such as polyethyleneglycol (PEG),^[^ ^32^, ^34^ ^]^ collagen,^[^ ^35^ ^]^ and derivatives of hyaluronic acid and alginate^[^ ^36^ ^]^ have been designed to undergo deformations without compromising cell viability. However, they typically present limitations such as single‐stage shape change (e.g., unidirectional bending/folding) and lack of controllability and reversibility over the shape manipulation. No work to date has been reported cytocompatible CHAs that enable complex multiple and reversible shape transformations in a programmed and/or “on‐demand” controllable manner for biomimicry of native tissue morphogenesis.

To address the aforementioned issues, we herein present biocompatible natural polymer‐based layered hydrogel systems capable of multiple different and/or reversible distinct shape changes over time via either preprogrammed design or user‐controlled environmental condition alternations. These layered hydrogels feature easy reproducible fabrication, cytocompatibility for cell encapsulation, shape controllability, multiple‐shape transformations over time, and tunable durations of different shape phases, and they may be broadly applicable as robust and versatile CHAs.

## 2 Results and Discussion

**Figure 1:**
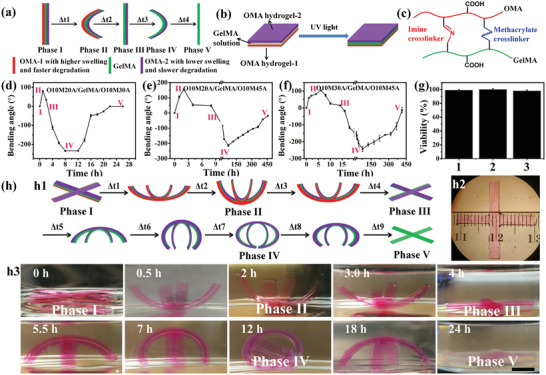
a) Schematic of the five‐phase transitions of a trilayer hydrogel bar. b) Sandwiching method to fabricate a trilayer hydrogel. c) Linkers between OMA and GelMA chains at the layer interface. d–f) The bending degree of the three trilayers as a function of time. g) Cell viability in the constructs of the three groups after shape evolution: 1) O10M20A/GelMA/O10M30A, 2) O10M20A/GelMA/O10M45A, and 3) O10M30A/GelMA/O10M45A. h) Cell‐laden four‐arm trilayer gripper and the programmable deformations: h1) schematic illustrating the entire process of shape evolution, h2) top view photomicrograph of a synthesized four‐arm gripper, and h3) photographs of four‐arm gripper shape changes over time. Scale bar indicates 0.5 cm. The two OMA layers were co‐crosslinked with methacryloxyethyl thiocarbamoyl rhodamine B (0.005%) for visualization. Data are presented as mean ± standard deviation (± SD), *N* = 3.

The overall strategy for a preprogrammed multiple‐shape morphing CHA is based on a trilayer approach depicted in **Figure** **1**a. The trilayer consists of two outer oxidized methacrylated alginate (OMA) layers with different swelling ratios and degradation rates and a GelMA (methacrylated gelatin) layer. This unique trilayer design is expected to undergo multiple‐shape (five‐phase) transformations during culture due to the swelling and degradation discrepancies of these layers. OMAs (O10M20A, O10M30A, and O10M45A) were synthesized by functionalizing alginate through both oxidation (10% oxidation) and methacrylation (20%, 30%, and 45%), and GelMA was synthesized by the reaction of type‐B gelatin with methacrylic anhydride (Table S1 and Figures S1–S4, Supporting Information). To fabricate the multiple‐step, preprogrammed shape morphing trilayer hydrogels, a “sandwich” method was used: GelMA solution containing live cells was placed between two prefabricated individual OMA layers and then crosslinked under UV light (Figure 1b and the Supporting Information). For the OMA layers fabrication, 30 s UV irradiation was applied to crosslink the OMA hydrogel precursor to form a stable hydrogel (Figure S5, Supporting Information) while at the same time preserving some unreacted methacrylate groups (Figure S6, Supporting Information) for subsequent adhesion to the GelMA layer. Then, 60 s UV irradiation was applied to crosslink the GelMA solution between the prefabricated OMA layers to fully crosslink the methacrylates in the GelMA (Figures S7 and S8, Supporting Information) and OMA layers to form a stable triple‐layered hydrogel. The working principle for the hydrogel layer interface adhesion lies in the formation of the crosslinks (Figure 1c, blue bond) through the photopolymerization of the remaining methacrylates in OMAs with the methacrylates in GelMA. In addition, the aldehyde groups on the OMA hydrogel surface react slowly with the amine groups on the GelMA hydrogel surface to form imine bonds, generating a second covalent bond, which further reinforces the interface adhesion (Figure 1c, red bond). As a result, the adhesion strength at the interface was similar to or even stronger than the ultimate tensile strength of the OMA and GelMA hydrogels alone (Figures S9 and S10, Supporting Information). This simple hydrogel coupling method makes it more adaptable and flexible compared to other routine methods, such as adhesion by addition of supramolecular glue,^[^ ^37^ ^]^ surface crosslinking by postsurface modification,^[^ ^38^ ^]^ and self‐curing of two independent layers,^[^ ^39^ ^]^ which typically require additional steps and longer time, making them time‐consuming and lower efficiency protocols.

To characterize the programmable actuation behaviors of the trilayer CHA, three trilayer hydrogel bars (O10M20A/GelMA/O10M30A, O10M20A/GelMA/O10M45A, and O10M30A/GelMA/O10M45A) encapsulating NIH3T3 fibroblasts in the GelMA layer were prepared and then cultured in growth media (GM) at 37 °C to investigate their shape changes over time. All three trilayer CHAs exhibited five programmable phase transitions (bidirectional bending) with distinct bending angles (the bending angle measurement calculation is described in Figure S11 in the Supporting Information), phase durations (Figure 1d–f; Figure S12, Supporting Information), and high cell viability (Figure 1g; Figure S13, Supporting Information). Since the bending of the trilayer constructs originates from the anisotropic swelling of the three hydrogel layers, the swelling properties of these hydrogels were examined to gain insight into the bending behaviors. It was found that the swelling of OMA hydrogels decreased with increased methacrylation (*S*_O10M20A_ > *S*_O10M30A_ > *S*_O10M45A_), and these OMA hydrogels exhibited much higher swelling ratios compared to the GelMA hydrogel (Figure S14a, Supporting Information). The dimensions of equilibrated hydrogels were consistent with the corresponding swelling ratios (Figure S14b, Supporting Information). This result indicates that within the trilayer system, the OMA hydrogel with the highest swelling ratio acted as an actuation layer while the GelMA and/or OMA hydrogel with a lower swelling ratio(s) acted as constraint layer(s). The transition of Phase I to Phase II was driven by the competitive swelling of the two OMA layers, and the degree of bending was determined by the swelling difference. Consequently, O10M20A/GelMA/O10M45A, which presented the largest swelling difference between two OMA hydrogels, generated the largest curvature in Phase II (Figure 1e). The competitive swelling and the unceasing but asynchronous degradation of the two OMA layers dominated the ensuing three steps and determined the entire timespan of the 5 phases. Since the OMA hydrogels degrade more rapidly with decreased methacrylation (O10M20A > O10M30A > O10M45A; Figure S15, Supporting Information), O10M20A/GelMA/O10M30A exhibited the shortest time period through all the phases. Since the degradation of the OMA layers over time simultaneously resulted in swelling^[^ ^40^ ^]^ and weakened the hydrogel mechanics,^[^ ^41^ ^]^ the ultimate bending arose from the net outcomes of the competition between these two factors. In comparison with the actuation OMA layers, the cell‐laden GelMA layer was very stable and showed minimal degradation (Figure S15, Supporting Information) during 4 weeks of culture. In addition, the Young's modulus and storage modulus of the cell‐laden layer minimally changed after the five‐phase transition (Figure S16, Supporting Information). Thus, an initially stable microenvironment was provided to the residing cells. With cell‐laden trilayered hydrogel actuators, we further fabricated more complicated 4‐arm gripper structures, which also showed five distinct phase transitions in a programmed manner (Figure 1h).

**Figure 2:**
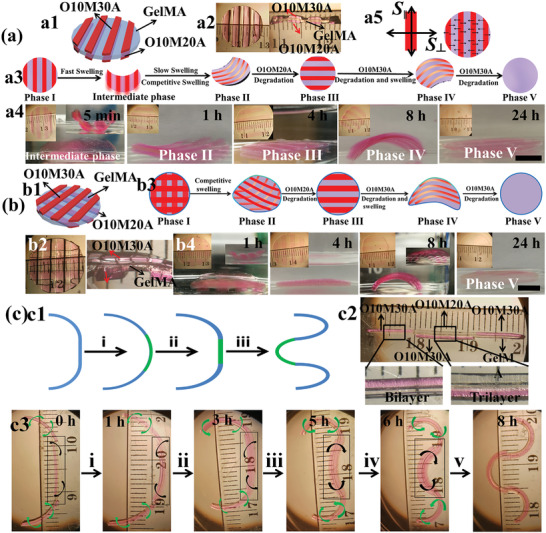
Cell‐laden “smart” trilayer hydrogel fabrication and the programmed deformation. a) Overlapping parallel‐strip patterns on both surfaces of a GelMA hydrogel: a1) schematic of the sample design, a2) photographs of top view and section view of a prepared sample, a3) schematic illustrating the entire process of the construct shape changes over time, a4) photographs of the construct actual shape changes over time, and a5) the speculated mechanism for the formation of an intermediate phase. b) Parallel‐strip patterns orthogonal to each other on both the surfaces of a GelMA hydrogel: b1) schematic of the sample design, b2) top view and section view photographs of a prepared sample, b3) schematic illustrating the entire process of the construct shape changes over time, and b4) photographs of the construct actual shape changes over time. All samples were cultured in GM at 37 °C, and GM was replaced with PBS before taking images for clarity. Insets in (a4) and (b4) on the top left corner show the constructs from the top view, and insets on the top right corner of some images (a4: 5 min; b4: 1 and 8 h) show the constructs from the side view. c) Biomimicry of branching morphogenesis: c1) schematic of branching morphogenesis of lung, step i: formation of a nascent bud, and steps ii and iii: cleft formation and terminal bifurcation; c2) photomicrograph of a typical cell‐laden discrete trilayer hydrogel bar designed to undergo branching morphogenesis; c3) photomicrographs of the 4D hydrogel system mimicking the process of lung branching morphogenesis by the discrete trilayer cultured in GM at 37 °C. Images were taken after replacing the GM with PBS for clarity. The two OMA layers were co‐crosslinked with methacryloxyethyl thiocarbamoyl rhodamine B (0.005%) for visualization. Scale bars indicate 4 mm.

Photolithography techniques offer powerful tools to incorporate antistrophic structures within a hydrogel with high precision, enabling complex shape transformation in a predesigned way.^[^ ^30^, ^42^ ^]^ Mask‐based photolithography allows facile patterning of OMA the GelMA hydrogel surface, and the design of the pattern enables unique control over preprogrammed CHA shape deformations. To demonstrate the feasibility of more sophisticated structure transformations with programmability, OMA‐patterned GelMA hydrogel disks showing parallel OMA strips on both surfaces (overlapping patterns (**Figure** **2**a1,a2) and perpendicular patterns (Figure 2b1,b2)) were fabricated. Unexpectedly, the overlapping patterned disk plunged into an intermediate phase where the disk bent perpendicularly with the long axes of the parallel strips instead of going directly to the expected Phase II (Figure 2a4, 5 min). This was likely due to the swelling in the transverse direction of the strips (*S*_┴_) exhibiting much greater strain than that from the longitudinal swelling (*S*_‖_) at the initial stage (Figure 2a5).^[^ ^10^ ^]^ Meanwhile, the gradually increasing *S*_‖_ along the long axis of the strip overcame the deformation in the intermediate phase, and thus the hydrogel sheet transformed to Phase II at 0.5 h. Then the subsequent phases occurred successively in a similar manner to that of the hydrogel bars and grippers. Interestingly, perpendicularly patterned hydrogel disks went through a five‐phase transition in a different manner (Figure 2b). The 3D structure in Phase II also originated from the competitive swelling of two OMA layers, and the disk bent along the longitudinal directions of the O10M20A strips, while the O10M30A strips stayed perpendicularly to the bending orientation. Once the bending force from the O10M20A strips was relieved by their degradation, the swelling of the O10M30A strips on the other side of the construct started reversing the bending from concave to convex and with the bending orientation perpendicular to the long axes of O10M30 strips (Phases III and IV). The construct then again resumed a flattened shape after degradation of O10M30A strips (Phase V). These results establish that by deliberately engineering the trilayer, complex structures may be obtained with multiple stages of deformation, which is beyond the capability of conventional shape‐morphing systems that only show unidirectional deformation.^[^ ^43^ ^]^

After having demonstrated this system can be designed to undergo multiple distinct different shape changes over time, we then wanted to explore its capacity to biomimic an actual developmental process such as branching morphogenesis. During development, branching morphogenesis is a pivotal process that occurs during the formation of many important organs/tissues, including the lung, kidney, salivary gland, and mammary gland.^[^ ^44^ ^]^ Some of the shape changes that take place in these organs/tissues are relatively similar. For example, branching morphogenesis of the lung occurs through the repeated formation of nascent buds and subsequent cleft creation and bifurcation (Figure 2c1).^[^ ^45^ ^]^ To mimic this process using our understanding of the programmable multiphase change behavior of the CHAs, cell‐laden discrete hydrogel bars were fabricated (Figure 2c2; Figure S17, Supporting Information), which consisted of two bilayers on both sides (O10M30A/GelMA) and a trilayer in the middle (O10M20A/GelMA/O10M30A). The morphogenetic process was then investigated in GM at 37 °C (Figure 2c3). The deformation started by the evagination of the middle segment (step i), which resembles the budding process. Upon the degradation of the O10M20A layer, the effect of the antagonistic strain of the O10M30A layer in the middle segment slowly emerged (step ii) and then dominated at 5 h (steps iii–v) to create a cleft‐like structure. The evolution of the tissue‐mimic continued by proceeding to form an invaginated basin accompanied with formation of two arcs on both sides after 8 h (step iv and v), which resemble two new “buds.” The increasing bending of the bilayers on both sides synchronized with the evagination and invagination, which further augmented the formation of the final bifurcated structure. The timing of each of these stages could be further controlled by changing the composition of the layer components to regulate their relative swelling and degradation rates. These findings demonstrate that this discrete multilayer hydrogel self‐deformed in a spatiotemporally programmed manner that enabled biomimicry of the important individual stages of branching morphogenesis.

**Figure 3:**
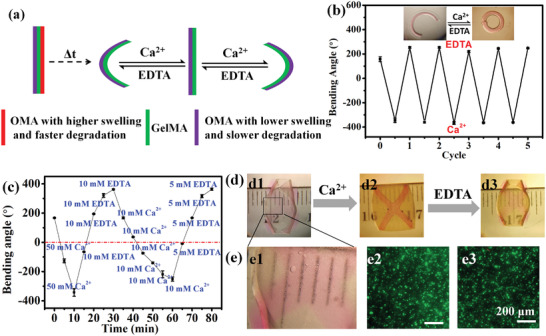
a) Schematic of proposed “on‐demand” reversible deformations of a bilayer derived from a trilayer by switching between exposure to Ca^2+^ and EDTA solutions. b) Cyclic reversible bending of O10M30A/GelMA bilayers due to alternating incubation in EDTA and Ca^2+^ solutions. Inset image shows the reversible bending of the hydrogel bar under alternating stimulations (transparent and red layers are the OMA layer and the GelMA layer, respectively). c) Shape manipulation of a cell‐laden bilayer by alternating Ca^2+^ and EDTA stimulation at 37 °C. d) Photomicrographs of the reversible 3D structure transitions of a quasi‐four‐petal flower by sequential treatment with Ca^2+^ (50 × 10^−3^ m, 10 min) and EDTA (5 × 10^−3^ m, 20 min) solutions. e1) Higher magnification images showing the cells in the GelMA layer and photomicrographs of live/dead stained cells e2) prior to and e3) after Ca^2+^ and EDTA treatment. Photos were taken after replacing the GM with PBS for clarity. Data are presented as mean ± SD, *N* = 3.

In addition to programmability, external “on‐demand” control over construct shape change may also be highly desirable for a cytocompatible hydrogel actuator as this would allow precisely defined and robust user‐regulated shape manipulation during the tissue formation process. Alginate and its derivatives form ionically crosslinked hydrogels with divalent cations such as calcium ions (Ca^2+^), and these crosslinks can be reversibly removed in the presence of chelating agents such as ethylene diamine tetraacetic acid (EDTA). The ionic crosslinking induces hydrogel network contraction while the removal of the cations results in hydrogel swelling relaxation. User‐controlled application of either ionic crosslinking or chelator solution permits regulation of construct environmental conditions and reversible deformation of layered hydrogel composites. Therefore, the bilayer obtained from the trilayer after the degradation of the fast‐degradation OMA layer offers a second opportunity to regulate the shapes of hydrogel actuators on demand (**Figure** **3**a). Taking this into consideration, the GelMA/O10M30A bilayer resulting from the quick degradation of the O10M20A layer in the O10M20A/GelMA/O10M30A trilayer CHA was utilized to verify the shape responses upon external environmental stimulation. By soaking the bilayer bar in solutions containing Ca^2+^ or EDTA, they completely changed bending directions (Figure 3b, inset). When the immersion solution was repeatedly switched, the hydrogels almost entirely recovered their previous shape for 5 cycles (Figure 3b; Figure S18, Supporting Information). Based on this property, an intelligent 4‐arm hydrogel that reversibly switched its shape with Ca^2+^ or EDTA alternations (Figure S19, Supporting Information) was fabricated to act as an environmentally controlled gripper for cargo transportation, as demonstrated by its ability to transfer an aluminum ball (0.2 g) from the Ca^2+^ solution to the EDTA solution (Figure S20, Supporting Information). Notably, the curvature of the cell‐laden bilayer could be readily tuned by varying the incubation time and/or the concentration of Ca^2+^/EDTA (Figure 3c). The bending rate highly depended on the concentration of Ca^2+^ (−55° and −25° min^–1^ with 50 × 10^−3^ and 10 × 10^−3^ m Ca^2+^, respectively), whereas the concentration of EDTA exerted no obvious influence on the bending rate (≈30° min^–1^ with both 10 × 10^−3^ and 5 × 10^−3^ m EDTA). Importantly, the cells inside the hydrogel remained highly viable after treating with both Ca^2+^ and EDTA (Figure 3e; Figure S21, Supporting Information). Furthermore, a cell‐laden 3D bilayer construct, designed to fold into the shape of “quasi‐four‐petal flower” via OMA surface‐patterning on a cell‐encapsulated GelMA hydrogel sheet (Figure S22, Supporting Information), bent reversely after treating with Ca^2+^ and reverted to the original shape after treating with EDTA (Figure 3d) while maintaining high cell viability (Figure 3e; Figure S23, Supporting Information). These results establish that the shape of cytocompatible CHAs can be modulated reversibly and repeatedly by external stimuli.

Currently, the majority of cell‐laden scaffold‐based tissue engineering strategies involve preparing, culturing and/or implanting a construct that does not change in shape over time, and providing instructive signals to the cells from the scaffold itself and/or the defect site to guide their differentiation and function.^[^ ^46^ ^]^ These geometrically static systems cannot directly contribute to the morphogenesis of complex tissue architectures. In addition to the drawbacks described earlier, most reported shape morphing hydrogel systems generally utilize differential swelling to evoke deformations in a short time frame.^[^ ^47^ ^]^ Therefore, they may be unsuitable for biomimicking native tissue developmental shape changes, where the shape evolution processes of tissue morphogenesis can occur over slow and/or fast time scales. Since our hydrogel actuators enable temporally controllable shape evolution in the presence of encapsulated cells, this system is well suited for morphodynamic tissue engineering applications. To slow down the shape morphing progress to a course of 3 or 4 weeks, the concentration of the OMAs was increased from 6% to 8%. The increased concentration diminished the hydrogel swelling (Figure S24, Supporting Information) and decelerated the degradation rate at the same time,^[^ ^48^ ^]^ resulting in protracted bending durations (Figure S25, Supporting Information).

**Figure 4:**
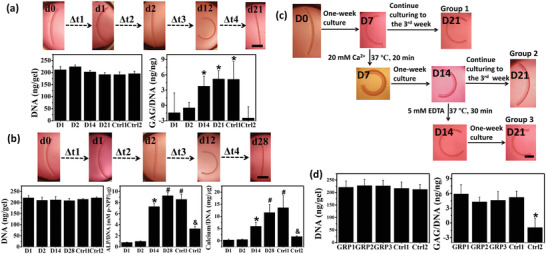
a) Programmable shape changes of trilayer CHA (O10M20A/GelMA/O10M45A) with encapsulated cells undergoing chondrogenesis and biochemical quantification of DNA content and GAG/DNA at each time point. *p < 0.05 compared to groups D1, D2, and Ctrl2. b) Programmable shape changes of trilayer CHA with encapsulated cells undergoing osteogenesis and biochemical quantification of DNA content, ALP/DNA, and calcium/DNA at each time point. ^*,#,&^ *p* < 0.05 compared to all groups with a different symbol or lacking a symbol. Ctrl1 stands for GelMA only hydrogel bar cultured in chondrogenic media at D21 (in (a)) or osteogenic media at D28 (in (b)), and Ctrl2 stands for trilayer hydrogel bar cultured in growth media at D21 (in (a)) and D28 (in (b)). c) Shape changes of GelMA/O10M45A bilayer derived from the O10M20A/GelMA/O10M45A trilayer during 3‐week culture in chondrogenic media: preprogrammed shape morphing (group 1), shape inversion at W1 by Ca^2+^ stimulation and subsequent culture to D21 (group 2), and shape recovery at W2 by EDTA and subsequent culture to D21 (group 3). d) DNA content and GAG/DNA ratios of all conditions at D21. Ctrl1 stands for GelMA only hydrogel bar cultured in chondrogenic media at D21. Ctrl2 stands for bilayer (GelMA/O10M45A) hydrogel bar cultured in growth media at D21. *p < 0.05 compared to all other groups. GRP1, GRP2, and GRP3 stand for group 1, group 2, and group 3, respectively. Black scale bars in the hydrogel photographs indicate 2 mm. Data are presented as mean ± SD, *N* = 3. Statistical tests were performed using one‐way ANOVA.

To demonstrate the capacity to control and change the shape of an engineered tissue during cell differentiation and new tissue formation, human mesenchymal stem cells (hMSCs) were seeded in the GelMA layer of the trilayer hydrogel bars (O10M20A/GelMA/O10M45A) and the constructs were cultured in chondrogenic and osteogenic media to induce chondrogenic and osteogenic differentiation of the cells, respectively (**Figure** **4**a,b). In both conditions, the trilayer CHAs programmably exhibited the five‐phase shape transitions during the culture period described previously (Figure 1a). The live/dead staining results indicated that the encapsulated cells maintained high viability (Figure S26, Supporting Information). Moreover, the production of a primary cartilage extracellular matrix component, glycosaminoglycan (GAG), during chondrogenesis and early and late osteogenic markers alkaline phosphatase (ALP) and calcium (Ca), respectively, during osteogenesis, all gradually increased over time and were significantly higher in comparison with the negative controls (trilayer hydrogel bars cultured in growth media at D21 or D28, Ctrl2). There was no statistical difference in DNA content between any of the groups or in differentiation marker expression between these experimental groups and positive controls (GelMA only hydrogel bar cultured in chondrogenic media at D21 or osteogenic media at D28, Ctrl1). These results indicate that the CHAs serving as a cell scaffold can change their shape in a programmable manner while not preventing encapsulated cell differentiation, and conversely cell differentiation did not prevent the hydrogel actuation.

To realize “on‐demand” shape controllability of the CHA system during cell differentiation and tissue regeneration, hMSC‐laden O10M45A/GelMA bilayer hydrogel bars derived from the O10M20A/GelMA/O10M45A trilayers after degradation of the O10M20A layer were cultured in chondrogenic media over 3 weeks. During culture in the differentiation media, shape changes with and without external stimulation over 3 weeks (21 days) were investigated (Figure 4c). The O10M20A layer in the O10M20A/GelMA/O10M45A trilayer completely disappeared after two‐day culture and the remaining bilayer further curled up to the GelMA side with continuously increasing curvature (group 1). The shape of the remaining bilayer could be tuned at any time point throughout the chondrogenesis process. For example, when the bilayer was treated with Ca^2+^ at week 1 (D7), this stimulus served to invert the orientation of the construct to curve toward the OMA side (group 2). This inversion could be reversed by EDTA stimulation at D14 (group 3). After treatment(s), the bilayers continued being cultured to D21. The inverted hydrogel (group 2) sustained its orientation despite some loss of the bending extent, and the recovered shape construct (group 3) stayed almost unchanged in its bending extent. Regardless of the external stimulation treatment, the cells remained highly viable (Figure 4d) and similar DNA levels were detected in all groups at D21 (Figure 4e). The production of the GAG was quantified to further assess the impact of the shape manipulation on chondrogenesis. The three experimental groups and the positive control group (GelMA only hydrogel bars cultured in chondrogenic media at D21, Ctrl1) exhibited similar amounts of GAG production to each other, and significantly more compared to the negative control group (GelMA/O10M45A bilayer hydrogel bars cultured in growth media at D21, Ctrl2). These results demonstrated that cells can be incorporated into the hydrogels and induced to differentiate, while at the same time, external stimuli may be applied to provoke multistage shape morphing of the constructs in an “on‐demand” manner. Alternative stimuli may be explored for a tissue such as bone, where Ca^2+^ production by cells might interfere with shape change and EDTA could impede osteogenesis.

## 3 Conclusions

In conclusion, this study demonstrated a potential strategy for “on‐demand,” multiple, and reversible shape morphing hydrogels using multilayered OMA and GelMA hydrogels. The simplicity, convenience, and strong adaptability of the fabrication methods make it simple to manufacture hydrogel actuators with various complexities. The CHAs transform into specific 3D architectures and undergo diverse alterations in either preprogrammed and/or user‐controlled manners with tunable phase durations. These CHAs can be designed to biomimic developmental and healing processes, such as branching morphogenesis, which may have great potential for constructing models of these processes and in tissue engineering applications. Such goals would be difficult to achieve with traditional systems due to limited deformation capacity, nonbiocompatible and/or cytotoxic materials and fabrication techniques, and harsh shape changing activation environments. This system provides a powerful tool and broadens the bioapplications of shape morphing hydrogels to investigate the role of multiple and reversible preprogrammed and “on‐demand” shape changes of extracellular matrix on cell behavior and tissue formation.

## 4 Experimental Section

For the experimental section, please refer to the Supporting Information.

## Conflict of Interest

The authors declare no conflict of interest.

## Supporting information

Supporting Information

## Data Availability

The data that support the findings of this study are available from the corresponding author upon reasonable request.
