# Supplementary material for: Cell‐Laden Multiple‐Step and Reversible 4D Hydrogel Actuators to Mimic Dynamic Tissue Morphogenesis
Source: Adv Sci (Weinh). 2021 Mar 1;8(9):2004616. doi: 10.1002/advs.202004616 (PMC8097354; doi:10.1002/advs.202004616)
Supplement: Supplementary file 1 — Supporting Information [file ADVS-8-2004616-s001.pdf]

WILEY-VCH

© 2021 Wiley-VCH GmbH

ADVANCED SCIENCE
Open Access

Supporting Information

for *Adv. Sci.*, DOI: 10.1002/advs.202004616

Cell-laden Multiple-Step and Reversible 4D Hydrogel Actuators to Mimic Dynamic Tissue Morphogenesis

Aixiang Ding, Oju Jeon, Rui Tang, Yu Bin Lee, Sang Jin Lee, and Eben Alsberg*

Supporting Information

Cell-laden Multiple-Step and Reversible 4D Hydrogel Actuators to Mimic Dynamic Tissue Morphogenesis

Aixiang Ding, Oju Jeon, Rui Tang, Yu Bin Lee, Sang Jin Lee, and Eben Alsberg*

1. Experimental:

1.1 Chemicals, instruments, and general methods

Unless specified, all solvents and reagents were used without further purification. Sodium alginate (**AL**, Protanal LF 20/40) was a generous gift from FMC Biopolymer. Bovine skin derived gelatin (type B), photoinitiator (2-Hydroxy-4'-(2-hydroxyethoxy)-2-methylpropiophenone, PI), Dulbecco's Modified Eagle Medium-High Glucose (DMEM-HG), Dulbecco's Modified Eagle Medium-Low Glucose (DMEM-LG), and fetal bovine serum (FBS) were purchased from Sigma. Dexamethasone was purchased from MP Biomedicals (Solon, OH). β-Glycerophosphate was purchased from CalBiochem. ITS^+ Premix and penicillin/streptomycin (P/S) were purchased from Corning Inc. (Corning, NY). Sodium pyruvate was purchased from HyClone Laboratories. Non-essential amino acid solution was purchased from Lonza Group (Basel, Switzerland). Ascorbic acid and ascorbic acid-2-phosphate were purchased from Wako Chemicals USA Inc. (Richmond, VA). Fibroblast growth factor-2 (FGF-2) was purchased from R&D Systems (Minneapolis, MN), transforming growth factor β1 (TGF-β1) was purchased from PeproTech (Rocky Hill, NJ), and bone morphogenetic protein-2 (BMP-2) was provided by Dr. Walter Sebald from the Department of Developmental Biology, University of Würzburg, Germany). *N*-(2-aminoethyl) methacrylate hydrochloride (AEMA) and methacryloxyethyl thiocarbamoyl rhodamine B (RhB) were purchased from Polysciences Inc., and other common chemicals, such as sodium peroxide, methacrylic anhydride, etc., were purchased from Fisher Scientific. ^1H NMR spectra were obtained on a 400 MHz Bruker AVIII HD NMR spectrometer equipped with a 5 mm SmartProbe™ at 25 ºC using deuterium oxide (D_2O) as a solvent and calibrated using (trimethylsilyl)propionic acid-d_4 sodium salt (0.05 w/v %) as an internal reference. DMEM-LG containing 0.05% PI (w/w) was used to dissolve the oxidized methacrylate alginate (OMA) and methacrylate gelatin (GelMA). DMEM-LG media was used to culture the hydrogels without cells. Cell growth media (GM) consisting of DMEM-LG (for NIH3T3 cell-laden hydrogels) or DMEM-HG (for stem cell-laden hydrogels) with 10% FBS and 1% P/S was used to culture the hydrogels with encapsulated cells. Hydrogel images were visualized using a Nikon SMZ-10 Trinocular Stereomicroscope equipped with a digital camera. A microplate reader (Molecular Devices iD5) was used to read data from the microplates.

1.2 Synthesis of OMAs and GelMA

OMAs with theoretical 10% oxidation degree and varying theoretical methacrylation degrees (20%, 30% and 45%) and GelMA with a theoretical 100% methacrylation degree were synthesized according to the reported literature.[S1] The O10M20A, for example, was synthesized with the following procedure: 10 g of sodium alginate was dissolved in 900 mL of diH_2O overnight, and 1.08 g of sodium periodate ($NaIO_4$) in 100 mL of diH_2O was rapidly added to the alginate solution under stirring in the dark at room temperature (RT). After

reaction for 24 h, 19.52 g of 2-ethanesulfonic acid (MES) and 17.53 g of sodium chloride (NaCl) were added, and the pH was adjusted to 6.5 with 5 N sodium hydroxide (NaOH). Then 1.77 g of *N*-hydroxysuccinimide (NHS) and 5.84 g of 1-ethyl-3-(3-dimethylaminopropyl)carbodiimide hydrochloride (EDC·HCl) were sequentially added to the mixture. After 10 min, 1.69 g of AEMA was added slowly. The solution was wrapped with aluminum foil to protect it from light and left to react for 24 h at RT. The mixture was then poured into 2 L of chilled acetone to precipitate out the crude OMA solid, which was further purified by dialysis against diH_2O over 3 days (MWCO 3.5 kDa, Spectrum Laboratories Inc.). The dialyzed alginate solution was collected, treated with activated charcoal (0.5 mg/100 mL, 50-200 mesh, Fisher) for 30 min, filtered through a 0.22 μm filter and frozen at -80 ºC overnight. The final O10M20A was obtained as white cotton like solid through lyophilization for at least 10 days. Other OMAs were synthesized through a similar procedure by adding different amounts of reactants and reagents, which, together with the actual methacrylation and actual oxidation, are shown in Table S1. Methacrylation degree was determined according to the method described in the literature.[S1] Briefly, the methacrylate modifications were quantified by comparing the methylene protons of methacrylate (~6.1 and 5.7 ppm) with the the proton integrals of the internal reference from the ^{1}H NMR spectra. The methacrylation degree (%) was defined as the number of methacrylated units per 100 repeating saccharide units. Oxidation degree was determined using a fluorescamine assay developed by modifying the TNBS (2,4,6-trinitrobenzene sulfonic acid) assay.[S2] Briefly, an excess amount of t-butyl carbazate (TBC, 0.1 mM in diH_2O) was used to react with each OMA solution (35.2 mg/mL in diH_2O) in diH_2O for 12 h at RT. The unreacted TBC was quantified using a fluorescamine solution (0.1 mM in DMSO). 10 μL of each sample was mixed with 10 μL of fluorescamine solution, followed by addition of 80 μL of DMSO and allowing for reaction at RT for 2 h. Fluorescence intensity at 470 nm was measured using the microplate reader under an exication of 390 nm. Formaldehyde was used as standard. The degree of oxidation (%) was defined as the number of oxidized units per 100 repeating saccharide units.

The GelMA was synthesized as follows: 20 g of gelatin was dissolved in 200 mL of PBS at 50 °C with stirring. 20 mL of methacrylic anhydride was added dropwise (1 mL/min) while vigorously stirring. The reaction was kept for 1 h at 50 °C with stirring and then at RT overnight. The reaction mixture was precipitated into excess acetone, dried in a fume hood and rehydrated to a 10 w/v % solution in diH_2O. The GelMA was purified by dialysis against diH_2O (MWCO 3.5 kDa) for 7 days at 50 °C to remove salts, unreacted methacrylic anhydride, and byproducts, and then filtered (0.22 μm filter) and lyophilized as described above.

Table S1. Feeding ratios of reactants and reagents for synthesis of OMAs and the actual methacrylation (%) of OMAs and GelMA and actual oxidation (%) of OMAs

**Polymer**	**Alginate (g)**	**$NaIO_4$ (g)**	**MES/NaCl (g/g)**	**NHS/EDC (g/g)**	**AEMA (g)**	**Actual methacrylation (%)**	**Actual oxidation (%)**
**O10M20A**	10	1.08	19.52/17.53	1.18/3.89	1.69	7.2	7.5
**O10M30A**	10	1.08	19.52/17.53	1.77/5.84	2.53	9.8	7.6
**O10M45A**	10	1.08	19.52/17.53	2.65/8.75	3.80	12.8	6.9
**GelMA**						80.0	

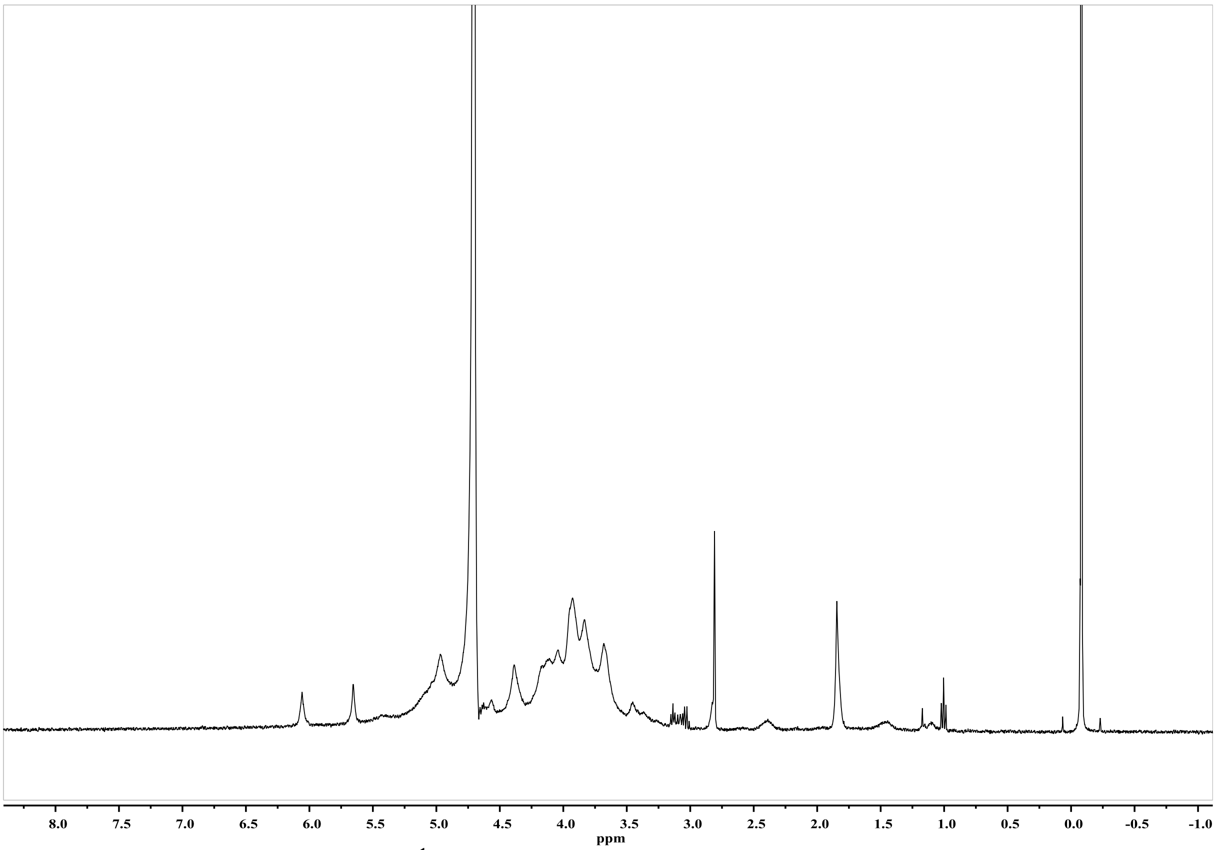

Figure S1. ^{1}H NMR of O10M20A in D_2O (2% w/w)

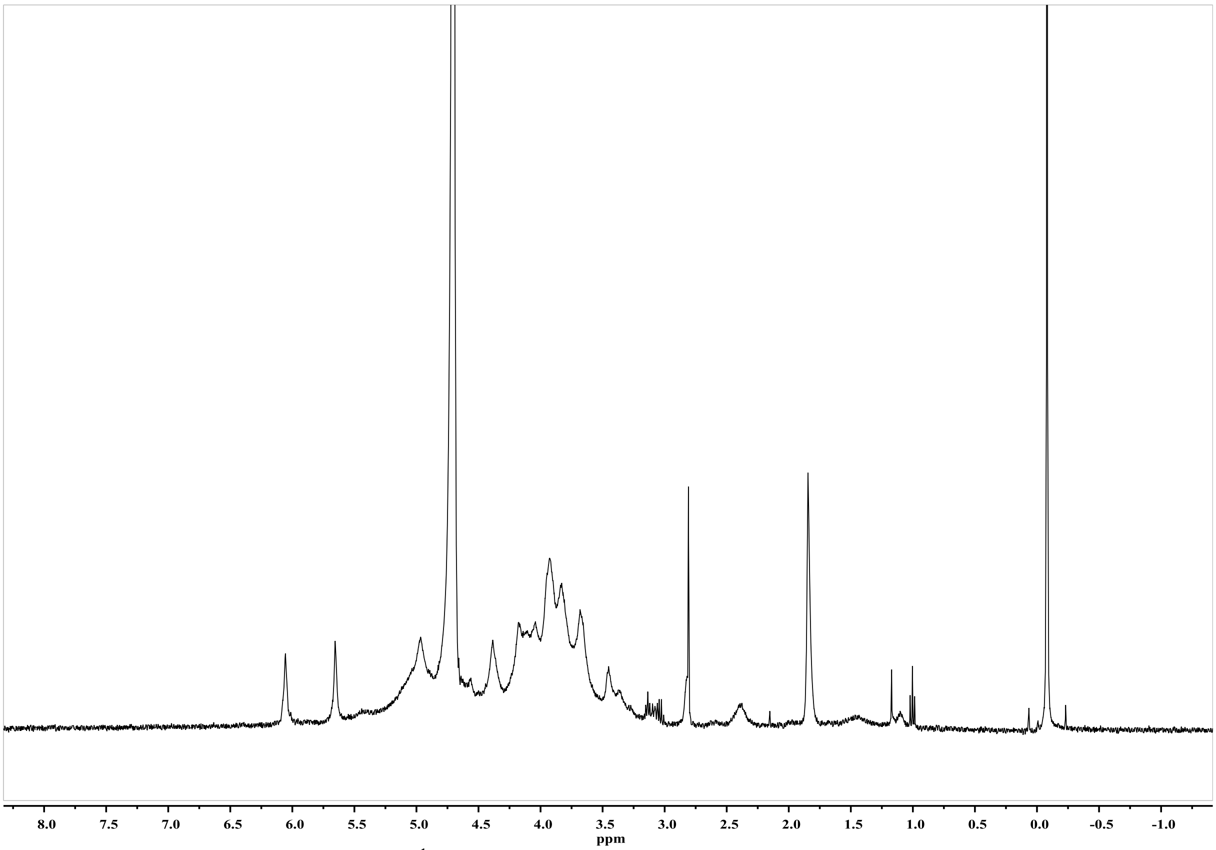

Figure S2. ^{1}H NMR of O10M30A in D_2O (2% w/w)

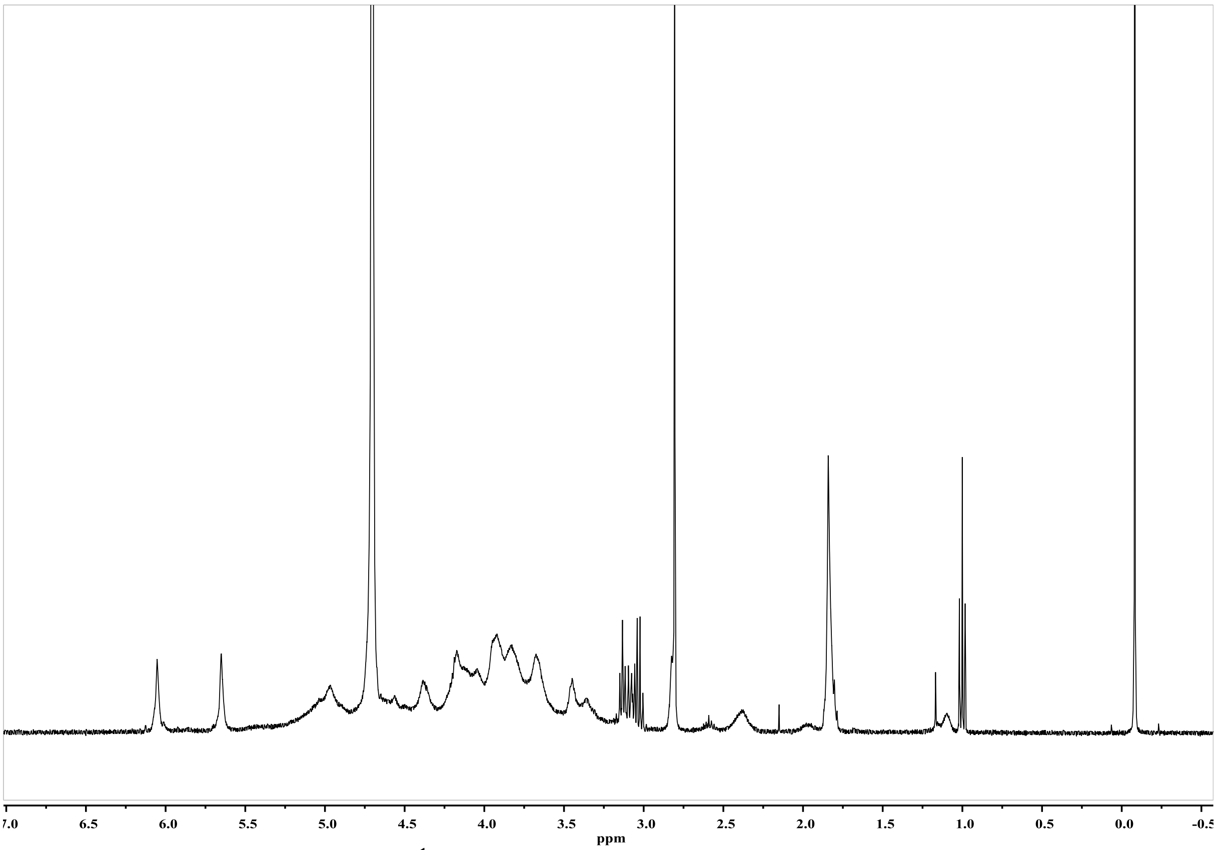

Figure S3. ^{1}H NMR of O10M45A in D_2O (2% w/w)

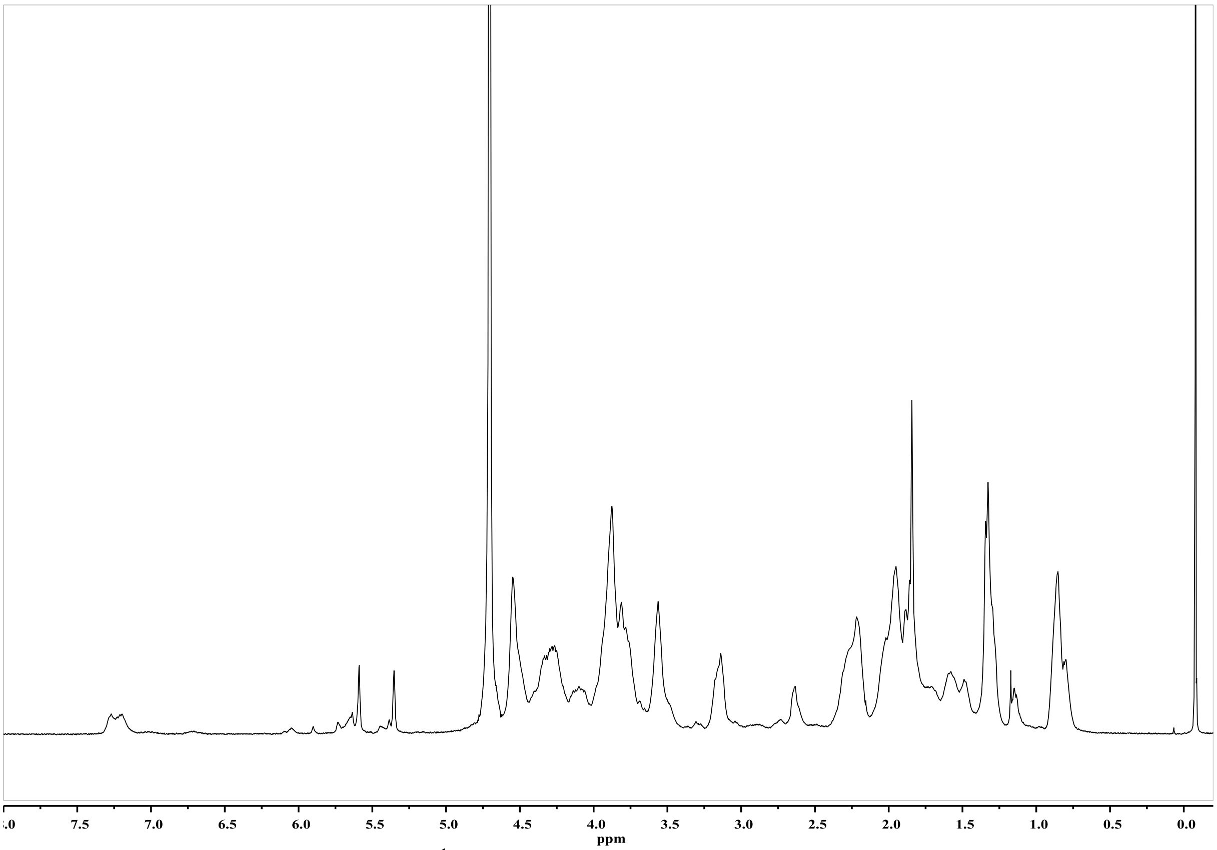

Figure S4. ^{1}H NMR of GelMA in D_2O (2% w/w)

1.3 Swelling and degradation tests

Solutions of OMAs (6%) in diH_2O and GelMA (14%) in DMEM-LG containing 0.05% PI were separately placed between two quartz plates with 0.75 mm spacers and UV crosslinked (320-500 nm, EXFO OmnicureR S1000-1B, Lumen Dynamics Group) at ~12 mW/cm^2 for 60 s to form the hydrogels. Then hydrogel disks were created using a biopsy punch (d = 6 mm). These hydrogel samples were frozen for 4 h at -80 ºC and lyophilized for 2 days. The masses of the dried gels were measured as initial weights (W_i). For the swelling test, the dried hydrogels were rehydrated by immersing into 1 mL of DMEM-LG and incubated at 4 °C to minimize the degradation for 10 h. The hydrogels were collected, and the swollen weights (W_s) were measured. The swelling ratios were calculated with the following equation: W_s/W_i (N = 3). For the degradation test, the dried hydrogels were incubated in 1 mL of DMEM-LG at 37 °C with media changes every other day to pre-determined time point. The rehydrated hydrogels were collected and dried by lyophilization to obtain dried mass (W_d). Mass loss was quantified as $(W_i-W_d)/W_i \times 100\%$ (N = 3) for each condition per time point.

1.4 Fabrication of cell-laden trilayer hydrogel bars

NIH3T3 fibroblast cells were cultured and expanded in NIH3T3 GM at 37 °C and 5 % CO_2 with media changes every 2 or 3 days. The cells were harvested for encapsulation when they reached 80% confluence.

To make the trilayer hydrogel bars, OMAs (6%) was dissolved in DMEM-LG and GelMA (14%) were dissolved in DMEM-LG containing NIH3T3 cells (5×10^6 cells/mL) and 0.05% PI. 200 μL of OMA solution was placed between two quartz plates with 0.4 mm spacers and subsequently photocrosslinked with UV light for 30 s to form an OMA hydrogel sheet. Trilayer hydrogel bars were fabricated with a "sandwich" method. Briefly, two single OMA hydrogel sheets (0.4 mm thickness) were made separately and 200 μL of the GelMA solution (14% in DMEM-LG containing 0.05% PI) with cells was placed between them. The "sandwich" was crosslinked by exposure to UV light for a further 60 s to create a trilayer hydrogel sheet, which was cut into trilayer hydrogel bars with dimensions of L × W × H = 13 × 2 × 1.2 mm (0.4 mm per layer). The trilayer hydrogel bars were immediately immersed in the NIH3T3 GM to record the corresponding shape changes.

To visually distinguish different hydrogel layers, 0.005% RhB was added to either an OMA layer or GelMA layer to impart the corresponding hydrogel with a red color.

1.5 Rheological properties test

All single layer hydrogels (cell-free OMA and cell-free/cell-laden GelMA hydrogels) were prepared 0.6 mm thick, and GelMA hydrogels in the tri-layer were fabricated with a thickness of 0.6 mm instead of 0.4 mm, while the outer OMA layer(s) were kept 0.4 mm thick to ensure the same degradation times for the outer OMA layers as those in triple layers described above (section 1.4). Hydrogels, (1) cell-free single layers (OMA hydrogels and GelMA hydrogels) for UV crosslinking time screening, (2) cell-laden GelMA single layers cultured in the GM for 2 h at 37 °C to represent the GelMA layers in the beginning phase (phase I), (3) triple layers for providing the cell-laden GelMA layer at final phase (phase V) after complete degradation of the two outer OMA layers, and (4) as-prepared single cell-free GelMA layers (GelMA w/o cells) for comparison, were made into 2 cm disks with a biopsy punch and punched again into small hydrogel disks with another biopsy punch (d = 8 mm) to test the rheological properties on a Kinexus Ultra+ rheometer (Malvern Panalytical). In oscillatory mode, a parallel plate (8 mm diameter) geometry measuring system was employed, and the gap was set to 0.6 mm. After each hydrogel disk was placed between the plates, all the tests were carried out at 25 °C. Oscillatory frequency sweep (0.1-100 Hz at 1% strain) tests were performed to measure storage moduli (G') and loss moduli (G"). N = 3.

1.6 Mechanical testing

The tensile testing was performed according to the reported literature using a mechanical testing machine (225lbs Actuator, TestResources, MN, USA) equipped with a 25 N load cell [S3] to evaluate the interfacial adhesive strength of the OMA and GelMA hydrogels. Briefly, the hydrogel samples (Figure S9) with an interfacial cross-sectional area of 5 × 1 mm^2 were attached to two hard paper backings using cyanoacrylate glue (Krazy Glue®, Elmer's Products Inc., Columbus, OH). The hard paper backings were then attached firmly with common commercial transparent tape to a "plastic loading platen", which was attached to the "load cell", and to a "sample cup" , which was fixed on the bottom platform of the mechanical testing machine with a 4 mm gap. The adhesion strength was determined by performing constant strain rate (1.25%/sec) tensile tests at room temperature (RT). The tests were performed no less than 3 times per group (N ≥ 3) and all the samples ruptured in the same relative location (i.e., O10M20A/GelMA and O10M45A/GelMA samples ruptured on the OMA sides, while O10M30A/GelMA ruptured at the interface). Representative stress-strain curves are shown in Figure S9 to demonstrate the sufficient interface adhesion. Three stress-strain curves from 3 different samples of each condition (OMAs, GelMA, and bilayers) are also shown in Figure S10.

For the compressive modulus tests, the cell-laden GelMA samples were prepared as described above (section 1.6, rheological test). The compressive moduli of the cell-laden GelMA layer (as-prepared single layer or obtained from the degradation of a trilayer) in the beginning and final phases were determined by performing uniaxial, unconfined constant strain rate (0.8%/sec) compression tests at RT on the mechanical testing machine. Note that the samples were compressed gently with a spatula to make a full contact with a common flat glass plate, and then the glassplate with the sample was loaded into the testing machine for the test. The Young's modulus of each sample was determined using the first non-zero slope of the linear region of the stress-strain curve within 0–10% strain (N = 3).

1.7 The bending angle measurement

The bending angle in this manuscript was defined according to previously reported literature.[S4] Briefly, the hydrogel curve (bended hydrogel bar) is extended to a circle that matches well with the hydrogel curvature using Image J software (Figure S11 and S18). The bending angle is determined by measuring the central angle generated by drawing two lines between the endpoints of the hydrogel curve and the circle center, respectively. The determination of bending angles of programmable trilayer hydrogel bars is shown in Figure S6, where the angle is positive when the trilayer bends toward the slower-swelling OMA layer side (purple layer), and it turns negative when the bending direction changes after the degradation of the outer faster-swelling OMA layer (red layer). The determination of "on-demand" bending angles of bilayer hydrogel bars is shown in Figure S18. The angle is positive when the bilayer bends toward the GelMA layer side, and it turns into negative when it bends toward the OMA layer side. N = 3.

1.8 Surface patterning of the cell-laden GelMA hydrogel

A photomask-based photolithography technique was adopted to pattern the cell-laden GelMA hydrogel surface with OMA hydrogel strips. To pattern OMAs onto the dual surfaces of a cell-laden GelMA hydrogel, a single surface patterned GelMA hydrogel (Scheme S1A) and a pre-patterned OMA layer (Scheme S1B) were fabricated separately. Briefly, to pattern OMA onto the single surface of a cell-laden GelMA hydrogel, 200 μL of GelMA solution (14% in DMEM-LG containing 0.05% PI) with NIH3T3 cells (5 × 10^6 cells/mL) was placed

between two quartz plates with a 0.4 mm spacer and UV crosslinked for 30 s. Then 200 μL of OMA solution (6% in DMEM-LG containing 0.05% PI) was placed onto the surface of the cell-laden GelMA hydrogel, which was covered immediately with another quartz plate. Then a photomask with a parallel strip pattern was attached onto the surface of the top quartz plate. After UV irradiation for 30 s, the uncrosslinked OMA solution was gently flushed using PBS (pH 7.4). To pattern OMA alone onto the quartz plate, 200 μL of OMA solution (6% in DMEM-LG containing 0.05% PI) was placed between two quartz plates with 0.4 mm spacers. Then a photomask with a strip pattern was attached onto the surface of the top quartz plate. After UV irradiation for 30 s, the top quartz plate was removed carefully and the uncrosslinked OMA solution remaining on the bottom quartz plate was gently flushed using PBS (pH 7.4). The two parts resulting from Scheme S1A and S1B were then aligned manually and then further crosslinked to form the final dual-surface patterned GelMA hydrogel (Scheme S1C and S1D). The dual-surface patterned hydrogels were then cropped into hydrogel disks using a biopsy punch (d = 15 mm) and immediately immersed into NIH3T3 GM to observe the shape changes.

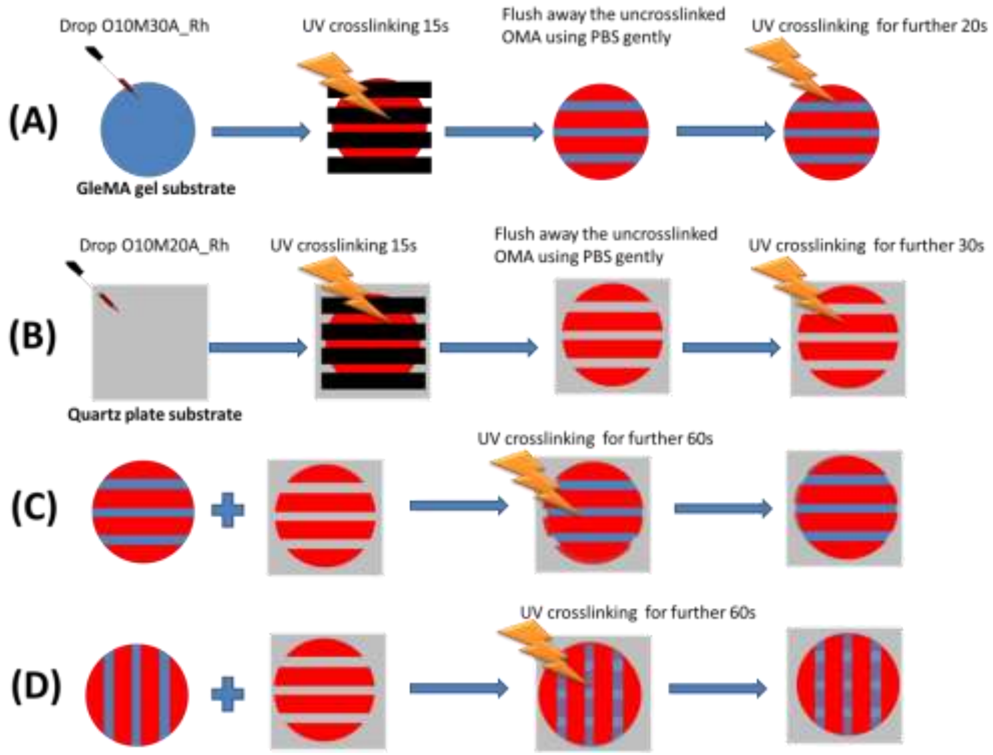

Scheme S1. Schematic illustration for patterning OMAs onto the surface of a GelMA hydrogel: (A) single GelMA surface patterning of OMA; (B) patterning OMA onto a quartz surface; formation of (C) parallel strips and (D) orthogonal strips on dual surfaces of GelMA hydrogel

1.9 Encapsulation of NIH3T3 and human mesenchymal stem cells (hMSCs) in GelMA hydrogel layer for cell-related experiments: live/dead staining, chondrogenesis and osteogenesis

In these studies, 0.005% RhB was incorporated into the OMA hydrogels to visually distinguish different hydrogel layers.

1.91 Cell expansion, encapsulation and cell-laden hydrogel incubation

NIH3T3 fibroblasts were expanded as described earlier in section 1.4 and encapsulated in the GelMA hydrogel layer to examine cell viability based on the live/dead staining assay at each predetermined time point during and/or after the hydrogel deformation. GelMA was dissolved in DMEM-LG (14%) containing NIH3T3 cells (5 × 10^6 cells/mL) and 0.05% PI.

Hydrogel bars or other constructs were produced as described earlier and cultured in NIH3T3 GM to investigate shape morphing and cell viability.

hMSCs were encapsulated in GelMA hydrogels (5×10^6 cells/mL) for both the chondrogenesis and osteogenesis studies. hMSCs from two different donors were used for the cell differentiation study. The hMSCs from donor 1 were isolated as described previously for the osteogenesis study.[S5] The hMSCs from donor 2 were isolated in the same manner but expanded in FGF-2 as described previously for the chondrogenesis study.[S6] hMSCs were expanded from passage 2 (P2) to passage 3 (P3) in the hMSC GM for osteogenesis or in hMSC GM containing 10 ng/mL FGF-2 for chondrogenesis in an incubator at 37 °C and 5 % CO_2 with media changes every 2 or 3 days. The cells were harvested for encapsulation when they reached 80% confluence. The cell-laden hydrogel bars or other constructs were fabricated as above. Hydrogels for chondrogenesis were cultured in basal pellet media (BPM) consisting of DMEM-HG with 1% ITS$^+$ Premix, 100 nM dexamethasone, 1 mM sodium pyruvate, 100 µM non-essential amino acids, 34.7 µg/mL ascorbic acid-2-phosphate and 1% P/S supplemented with 10 ng/mL TGF-β1. Hydrogels for osteogenesis were cultured in osteogenic media (OM) consisting of DMEM-HG with 10% FBS, 1% P/S, 10 mM β-glycerophosphate, 50 µM ascorbic acid, and 100 nM dexamethasone supplemented with 100 ng/mL BMP-2. All these hydrogels were cultured in 12-well tissue culture plates filled with 2 mL of culturing media and placed in a humidified incubator at 37 °C with 5% CO_2 for 3 (chondrogenesis) or 4 (osteogenesis) weeks with media changes every 2 days.

1.92 Live/dead staining

A live/dead staining assay was carried out to examine the viability of encapsulated cells at each designated time point using fluorescein diacetate (FDA, Sigma), which stains the cytoplasm of viable cells green, and propidium iodide (PI, Sigma), which stains the nuclei of dead cells red. 1 mL of live/dead staining solution, which was freshly prepared by mixing 8 µL of FDA solution (5 mg/mL in DMSO) and 8 µL of PI solution (2 mg/mL in PBS, pH 7.4) with 5 mL of PBS (pH 8.0), was added to each well containing the cell-hydrogel constructs. After 5 min incubation at RT in the dark and washing with 1 mL of PBS two times, *z*-stacked fluorescence images of the samples were taken using an ImageXpress Pico Automated Cell Imaging System equipped with a 5 mega Pixel CMOS digital camera (Molecular Devices). The individual *z*-stacked images were assembled using CellReporterXpress software (Molecular Devices). Quantification of the cell viability was based on the live/dead staining images, in which the green staining and red staining represented live cells and dead cells, respectively. Cell counts were carried out using Image J software (NIH).[S7] Cell viability was calculated as follows: (number of green (live) stained cells)/(number of green + red stained cells) × 100%. Ten random fields (1.8 cm × 2.3 cm) from 3 images of 3 samples were selected for each group.[S8]

1.93 Biochemical quantification of chondrogenesis and osteogenesis

To investigate the osteogenic and chondrogenic differentiations of hMSCs within the GelMA layer of the trilayer hydrogel bars, hydrogel actuators were cultured as described in section 1.91, removed from the plates at predetermined time points (d1, d2, d14 and d21 for the chondrogenic differentiation of the trilayer hydrogel bars, and d1, d2, d12 and d28 for the osteogenic differentiation of the trilayer hydrogel bars) and stored at -20 ºC until all samples were collected. The chondrogenic hydrogels were put in 0.6 mL of papain buffer (Sigma) and the osteogenic hydrogels were put in 0.5 mL of CelLytic^{TM} buffer (Sigma), and these hydrogels were then homogenized at 35,000 rpm for 2 min using a TH homogenizer (Omni International) on ice. The samples for chondrogenic studies were digested at 65 ºC for 24 hours and centrifuged for 10 min at 15,000 rpm, and then the supernatants were collected for

DNA and glycosaminoglycan (GAG) quantifications (N=3). The samples for the osteogenic study were centrifuged for 5 min at 500 g and 4 ºC, and the samples were collected for DNA, alkaline phosphatase (ALP), and calcium analysis (N=3).

Per manufacturer's instructions, a Picogreen assay kit (Invitrogen) was used to quantify the DNA content in the supernatant. Fluorescence intensity of the dye-conjugated DNA solution was measured using a microplate reader with an excitation of 480 nm and emission of 520 nm.

The GAG content was quantified using a DMMB (1,9-dimethylmethylene blue) assay.[S9] Briefly, DMMB dye solution was prepared by dissolving 21 mg of DMMB and 2 g of sodium formate in 5 mL of absolute ethanol, and then 795 mL of diH_2O was added to the solution to reach a total volume of 800 mL. The pH of the solution was adjusted to 2 using formic acid. Then diH_2O was added to the solution again to bring the solution to a total volume of 1000 mL. For GAG quantification, 40 μL of supernatant from the digested samples was transferred into 96-well plate, to which 125 μL of DMMB solution was then added. Absorbance at 595 nm was recorded on a microplate reader. GAG content was normalized to DNA content.

For ALP quantification, 100 μL of supernatant was treated with *p*-nitrophenylphosphate ALP substrate (pNPP, 100 μL, Sigma) at 37 °C for 30 min, and then 0.1 N NaOH (50 μL) was added to stop the reaction. The absorbance was measured at 405 nm using a microplate reader. To quantify the calcium content, an equal volume of 1.2 N HCl was added into each remaining lysate solution and pellet, and then the mixed solutions were centrifuged at 500 g for 5 min at 4 ºC. A calcium assay was then performed using a kit (Pointe Scientific) per the manufacturer's instructions. Briefly, supernatant (4 μL) was mixed with a color and buffer reagent mixture (250 μL), and the absorbance was recorded at 570 nm on a microplate reader. All ALP and calcium content measurements were normalized to DNA content.

2.0 Statistical analysis

Statistical analysis was performed with one-way analysis of variance (ANOVA) with Tukey honestly significant difference post hoc tests using Origin software (OriginLab Corporation, MA). A value of $p < 0.05$ was considered statistically significant. The sample sizes for data quantification are indicated in the corresponding experimental sections and figure legends. All quantitative data was expressed as mean ± standard deviation (± SD).

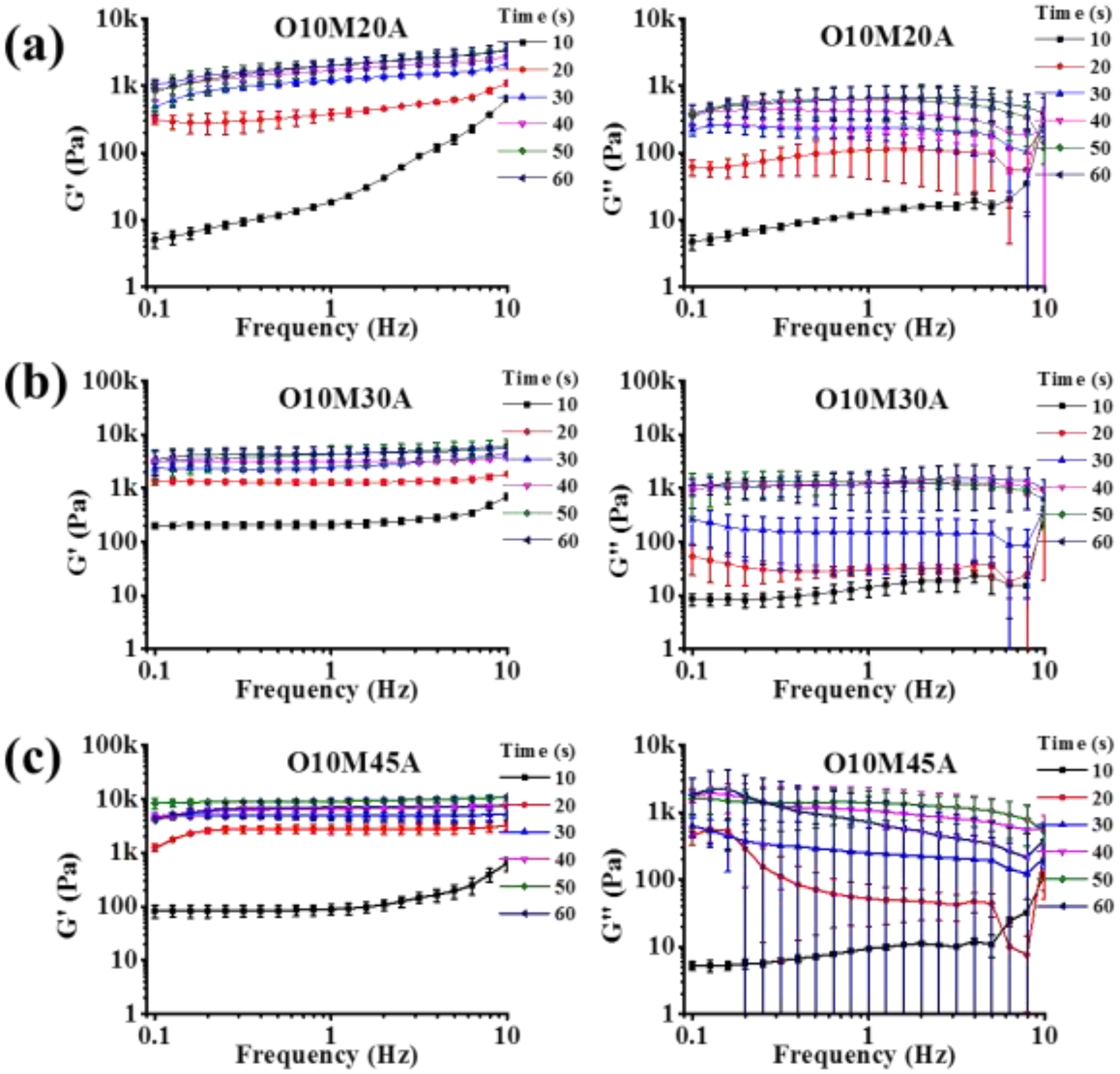

Figure S5. The effect of UV crosslinking time on the storage and loss moduli of hydrogels formed from (a) O10M20A, (b) O10M30A, and (c) O10M45A. Data are presented as mean ± SD, $N = 3$.

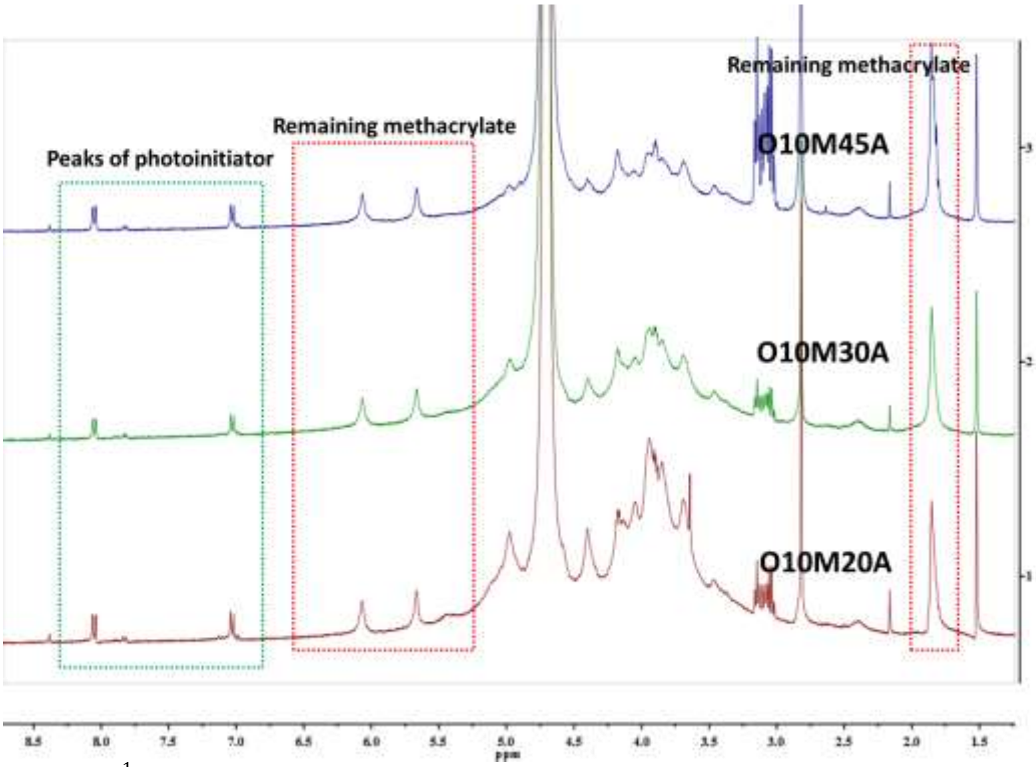

Figure S6. ^{1}H NMR spectra showing the remaining methacrylate after 30 s of photocrosslinking O10M20A, O10M30A, and O10M45A.

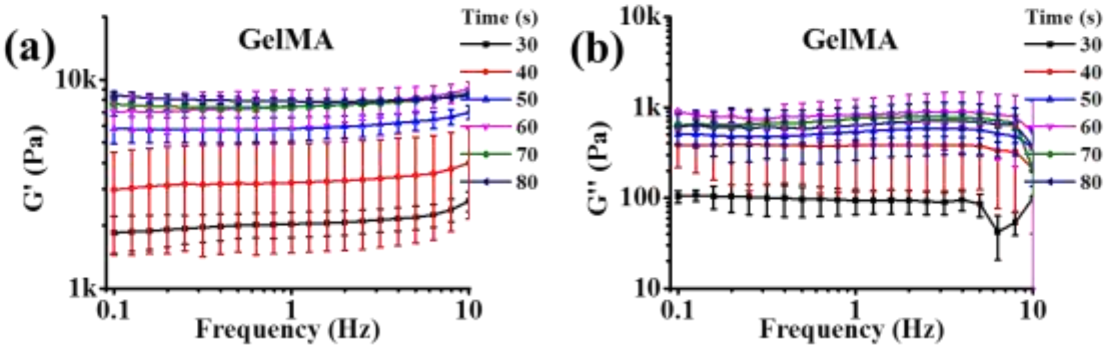

Figure S7. The effect of UV crosslinking time on the (a) storage and (b) loss moduli of GelMA hydrogels. G’ and G’’ remained relatively unchanged after 60 s UV crosslinking, suggesting complete crosslinking. Data are presented as mean ± SD, *N* = 3.

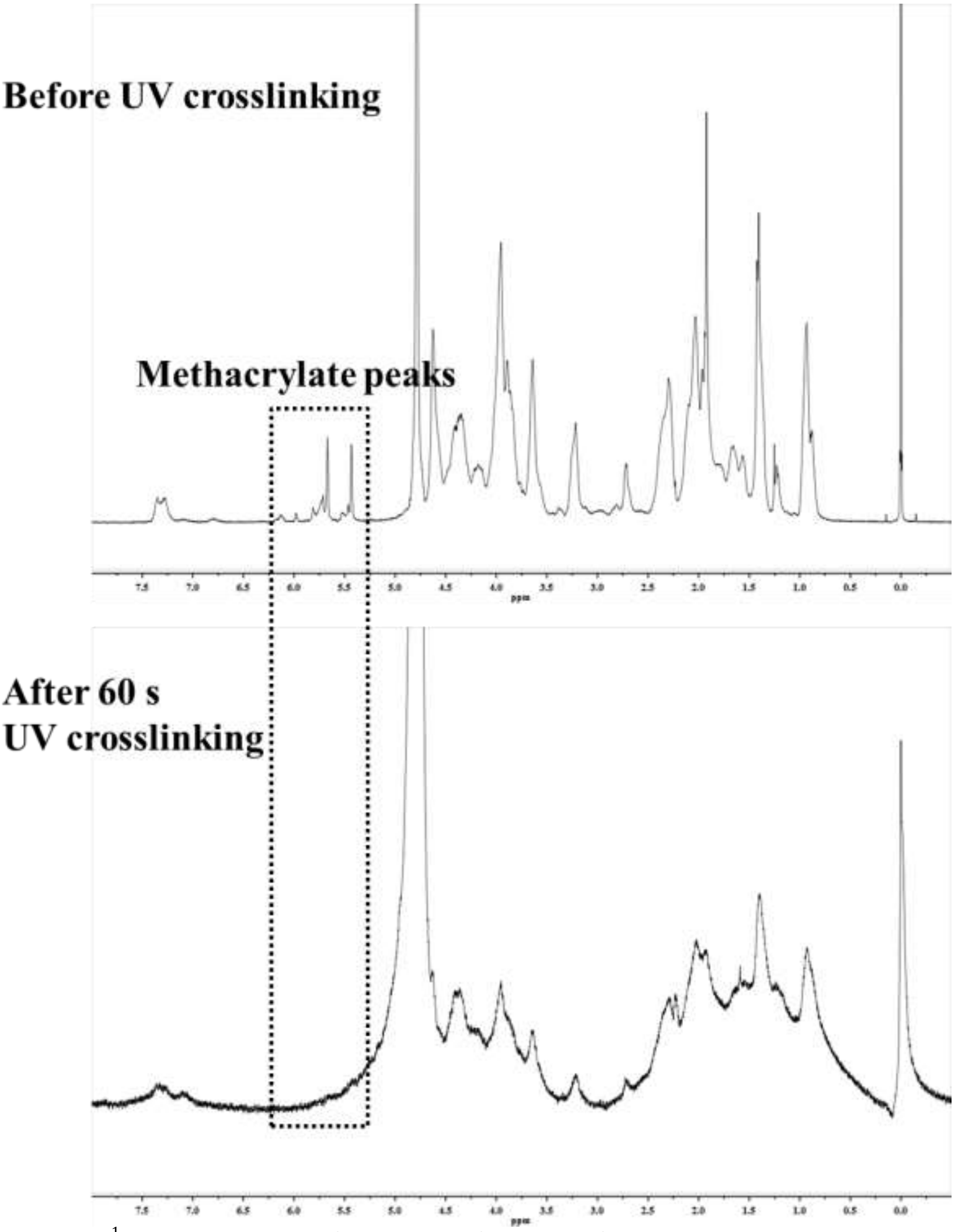

Figure S8. ^{1}H NMR spectra of GelMA before and after 60 s UV crosslinking. The disappearance of methacrylate peaks (5.3 ~ 5.8 ppm) indicates complete consumption of methacrylate groups.

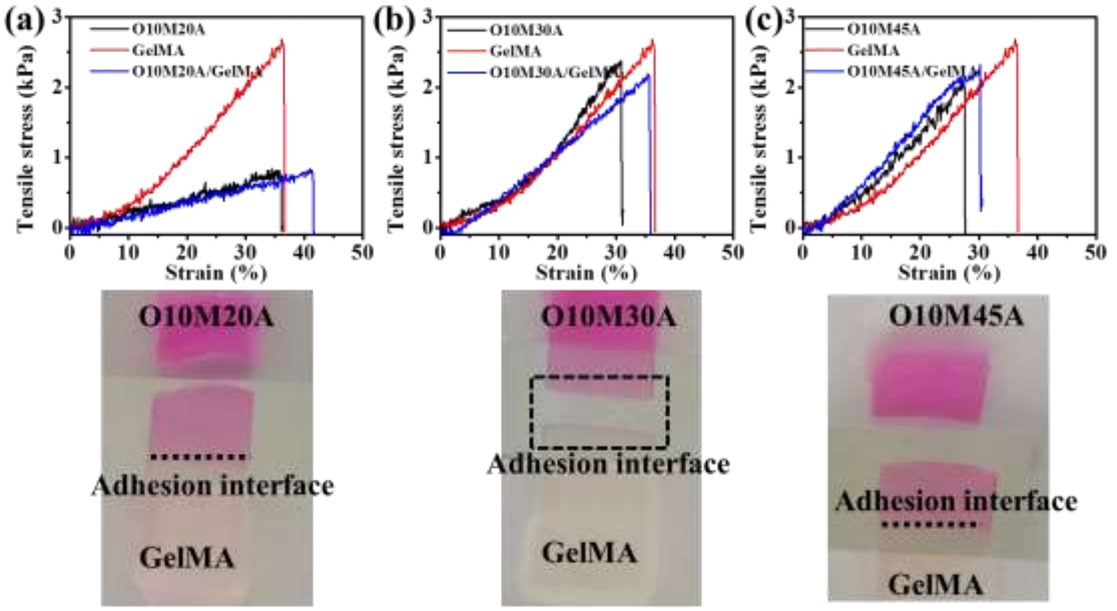

Figure S9. Stress-strain plots from representative tensile tests demonstrating strong interfacial adhesion of (a) O10M20A/GelMA, (b) O10M30A/GelMA, and (c) O10M45A/GelMA. The interfacial adhesion strengths of O10M20A/GelMA and O10M45A/GelMA are stronger than the ultimate tensile strength of corresponding OMA hydrogels, therefore the hydrogel strips ruptured on the OMA side. The interfacial adhesion strength of O10M30A/GelMA is weaker than the ultimate tensile strength of the two individual hydrogels (O10M30A and GelMA), and the hydrogel strips ruptured at the interface. This finding is associated with the competition between the increase in tensile strengths of the OMA hydrogels with the increase of the OMA/GelMA interfacial adhesion strengths in relation to the increasing OMA methacrylation. For the O10M30A/GelMA bilayer, the increase of the interfacial adhesion strength lagged behind the increase of the O10M30A hydrogel tensile strength. Therefore, this bilayer ruptured at the interface. In contrast, the increase of the interfacial adhesion strength of the O10M45A/GelMA bilayer surpassed the increase of the O10M45A hydrogel tensile strength. Therefore, this bilayer ruptured on the O10M45A side instead of at the interface.

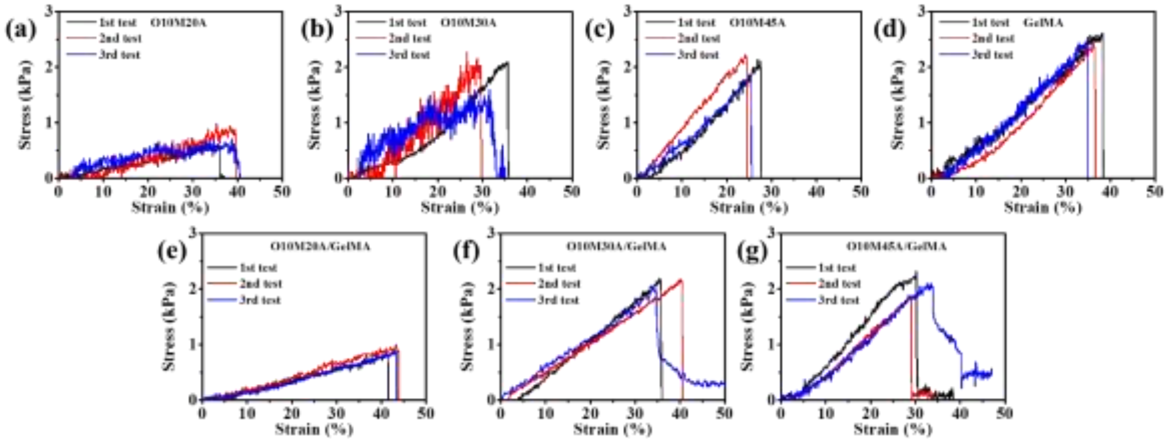

Figure S10. Stress-strain plots from tensile tests of 3 independent samples of (a) O10M2OA, (b) O10M30A, (c) O10M45A, (d) GelMA, (e) O10M20A/GelMA, (f) O10M30A/GelMA, (g) O10M45A/GelMA.

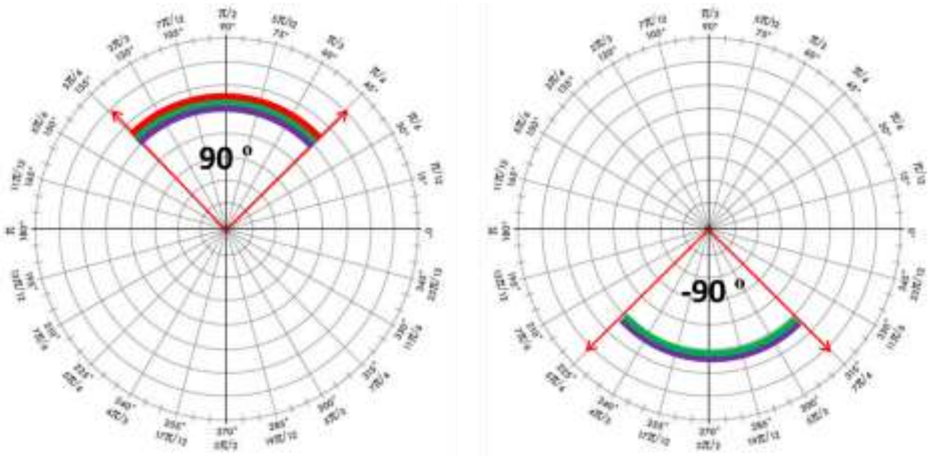

Figure S11. Pictorial depiction of the definition of bending angle measurement for the programmable shape change of trilayer hydrogel bars. The bending angle is positive when the trilayer bends toward the slower-swelling OMA side (purple layer), and it becomes negative when the bending direction changes after the degradation of the outer faster-swelling OMA layer (red layer).

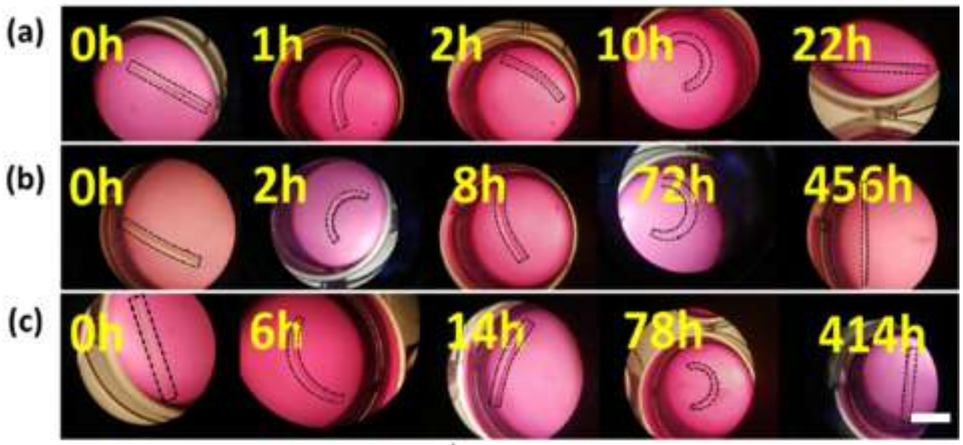

Figure S12. Representative photomicrographs showing the actual shape changes of the trilayer constructs overtime: (**a**) O10M20A/GelMA/O10M30A, (**b**) O10M20A/GelMA/O10M45A, and (**c**) O10M30A/GelMA/O10M45A. Methacryloxyethyl thiocarbamoyl rhodamine B (RhB, 0.005%) was crosslinked within the OMA layer in red font to aid in visualization. The dotted outlines indicate the shape of the hydrogel strips. Scale bar: 5 mm.

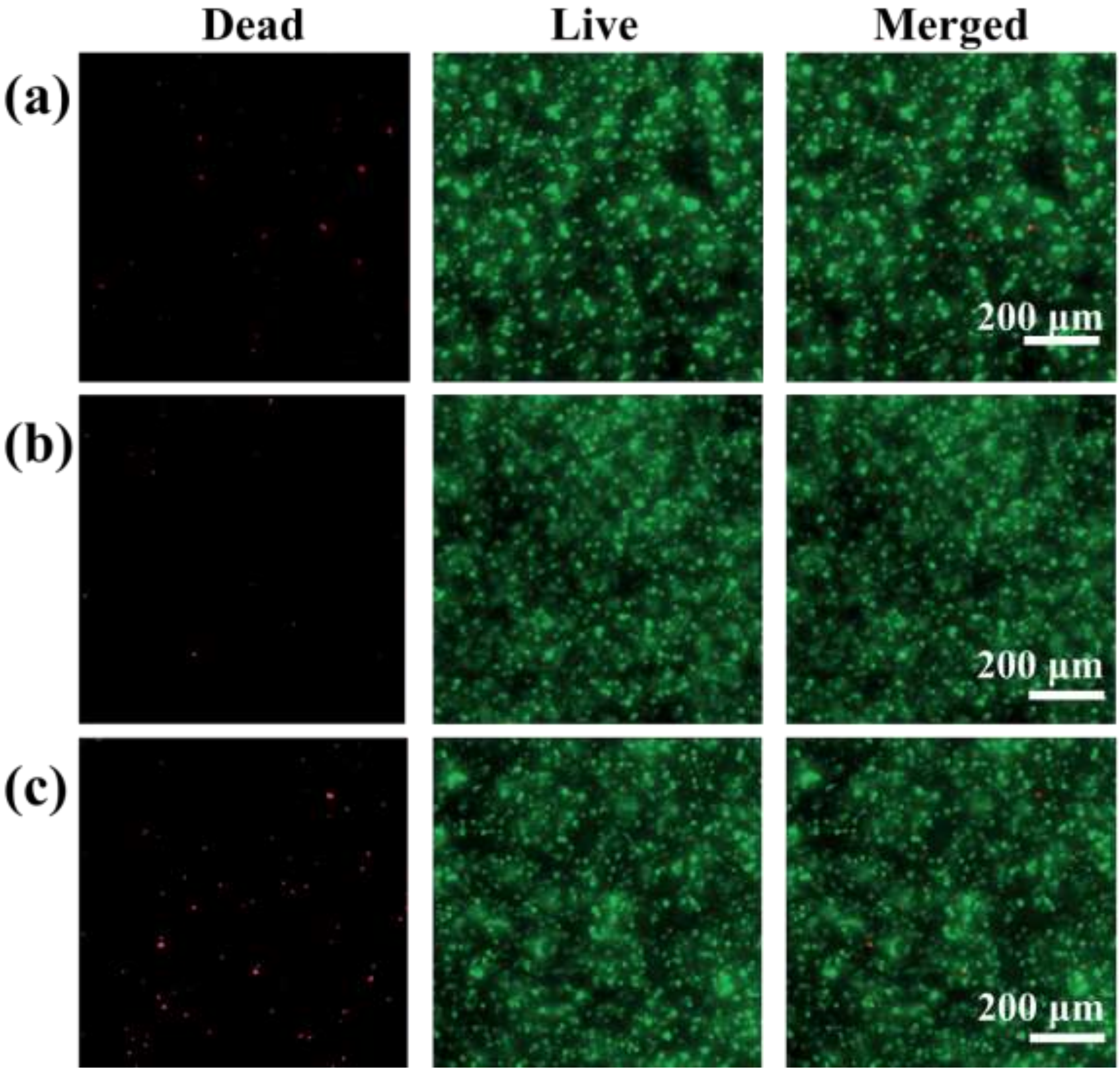

Figure S13. Representative photomicrographs of live/dead stained NIH3T3 cells encapsulated in the hydrogels after the five-phase transitions: (**a**) O10M20A/GelMA/O10M30A, (**b**) O10M20A/GelMA/O10M45A, and (**c**) O10M30A/GelMA/O10M45A.

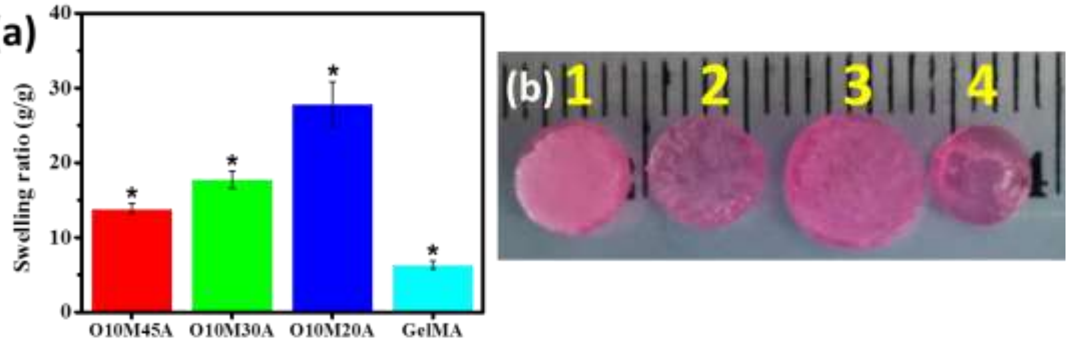

Figure S14. (a) Swelling ratios of single layer OMA hydrogels and GelMA hydrogel in DMEM-LG at 4 ºC after 10 hours, *$p < 0.05$ compared to all other groups (one-way ANOVA); (b) Photographs of hydrogels after equilibrated swelling in DMEM-LG: (**1**) O10M45A, (**2**) O10M30A, (**3**) O10M20A, and (**4**) GelMA. Data are presented as mean ± SD, $N = 3$.

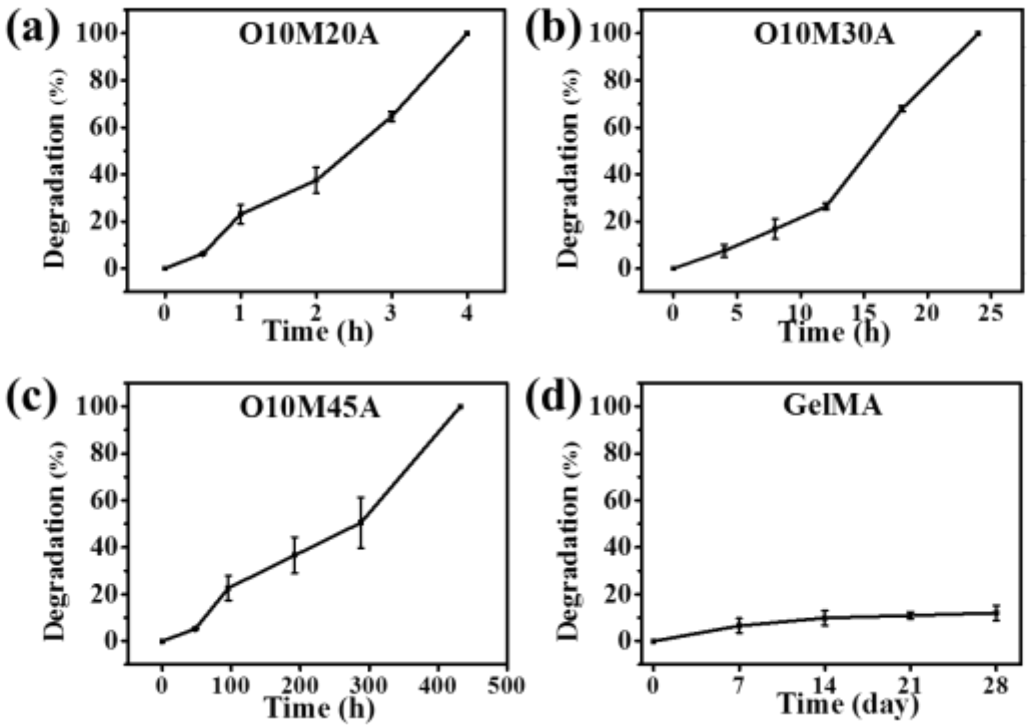

Figure S15. Degradation profiles of hydrogels in DMEM-LG at 37 °C: (a) O10M20A, (b) O10M30A, (c) O10M45A, and (d) GelMA. Data are presented as mean ± SD, $N = 3$.

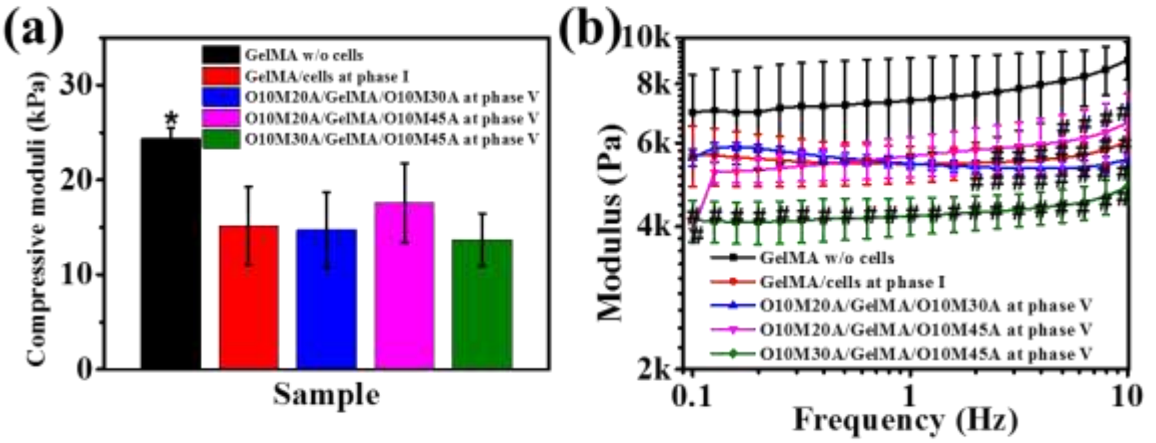

Figure S16. (a) Compressive moduli and (b) storage moduli of as-prepared cell-free GelMA hydrogels (GelMA w/o cells) and cell-laden GelMA hydrogels at phases I (GelMA/cells at phase I) and V (cell-laden GelMA obtained from the respective triple layer, i.e., O10M20A/GelMA/O10M30A at phase V, O10M20A/GelMA/O10M45A at phase V, and O10M30A/GelMA/O10M45A at phase V). Loading of cells decreased the modulus and the modulus changed minimally after the 5-phase transition, $*p < 0.05$ compared to other groups, #p < 0.05 compared to GelMA w/o cells group at the same frequency point. Data are presented as mean ± SD, $N = 3$. Statistical tests were performed using one-way ANOVA.

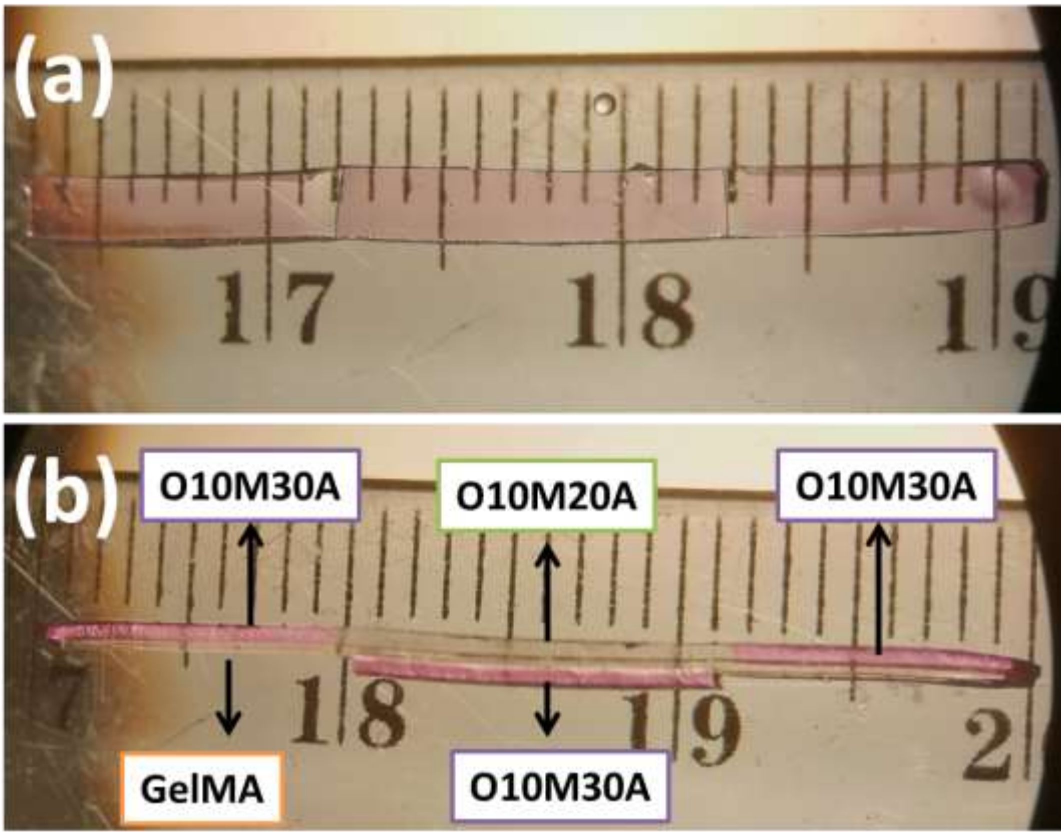

Figure S17. Photomicrographs of (a) top and (b) side views of a discrete trilayer hydrogel bar.

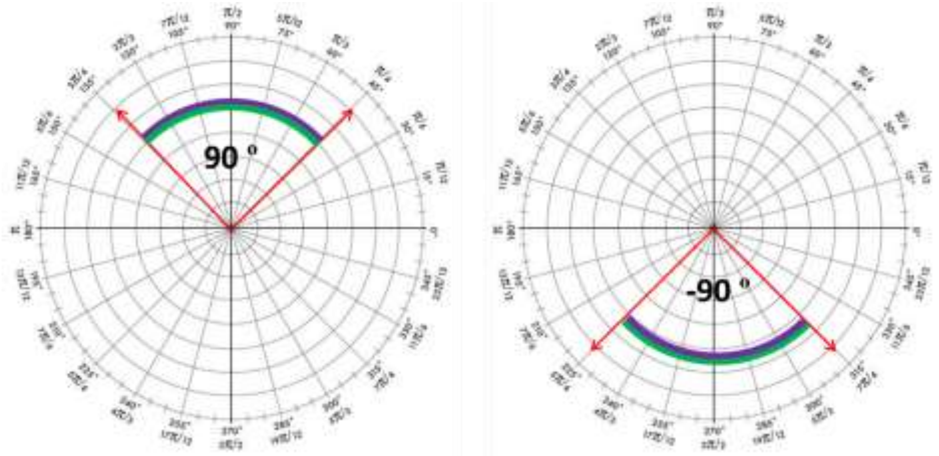

Figure S18. Pictorial depiction of the definition of bending angle measurement for the "on-demand" shape changes of bilayer hydrogel bars. The bending angle is positive when the bilayer bends toward the GelMA side (green layer), and it becomes negative after changing its bending direction toward the OMA side (purple layer).

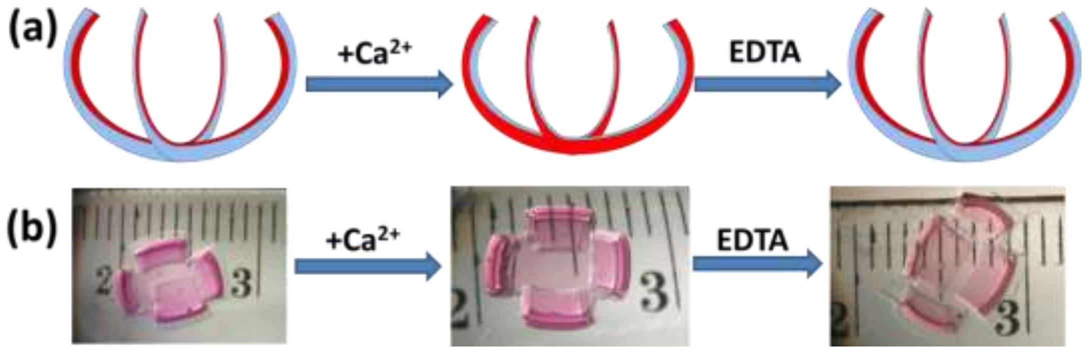

Figure S19. (a) Schematic illustration and (b) the actual protographic images of the reversible shape switching of a 4-arm bilayer gripper upon alternating Ca^{2+}/EDTA treatment. The blue (in (a)) / transparent (in (b)) and red layers are the OMA and GelMA layers, respectively.

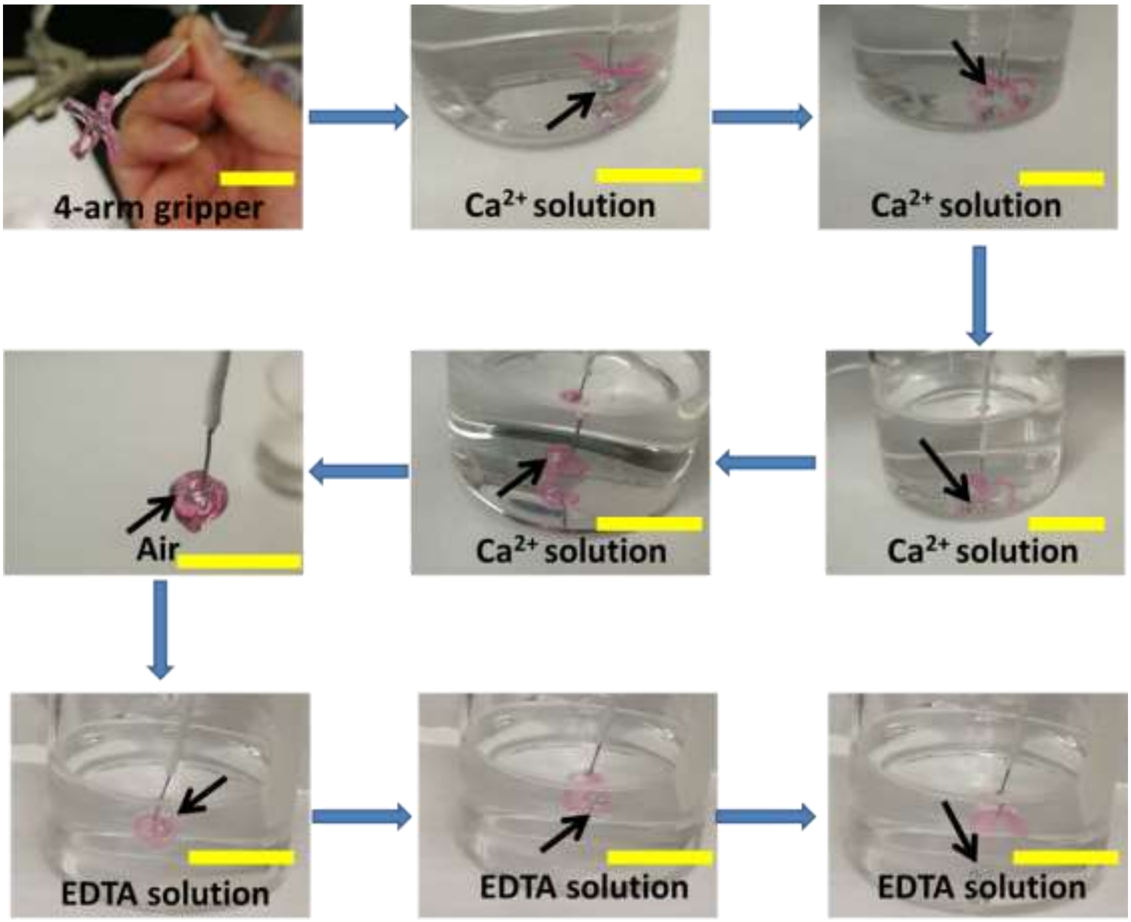

Figure S20. The application of the 4-arm gripper for transferring an aluminum ball (0.2 g). Scale bars indicate 2 cm.

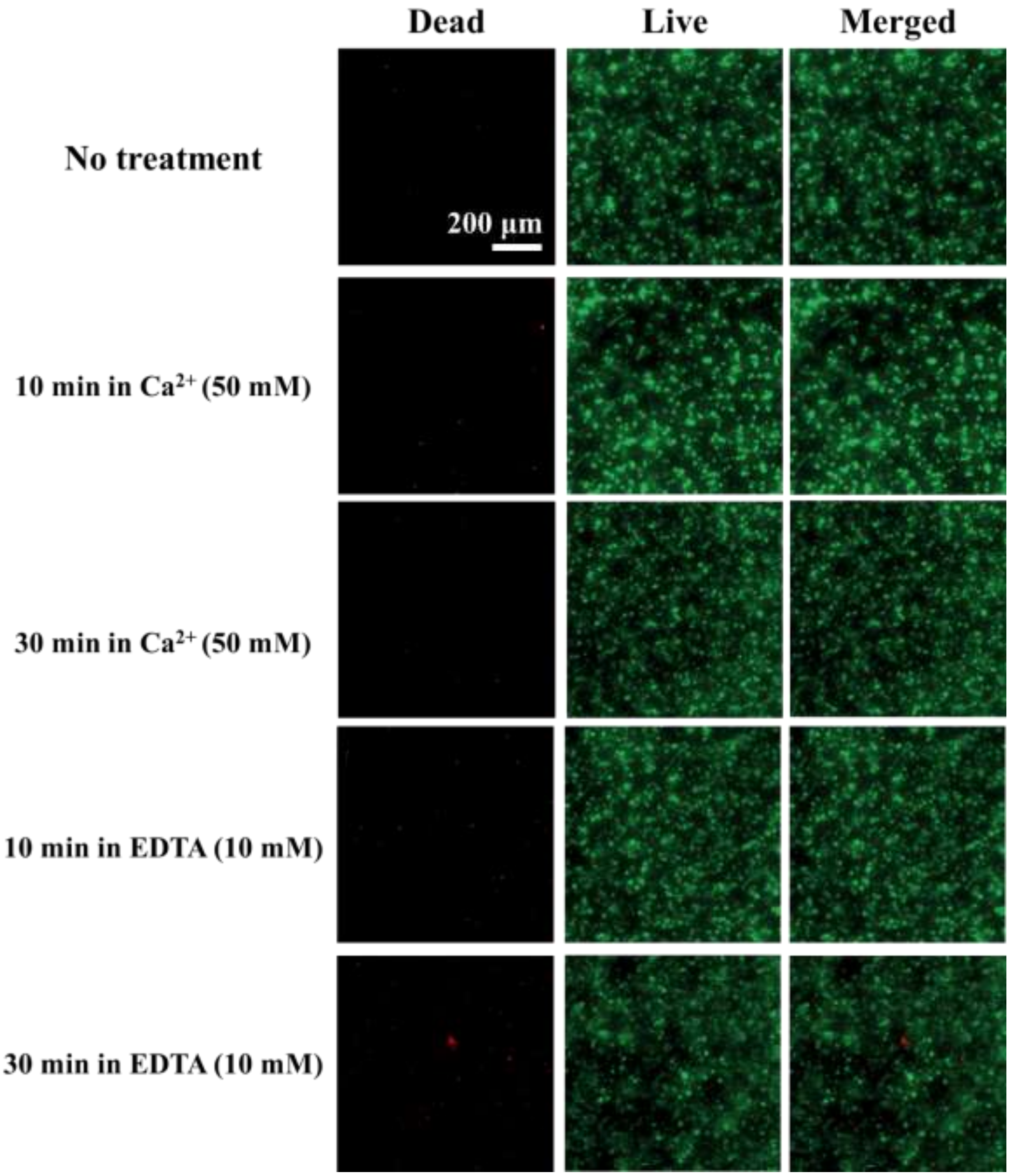

Figure S21. Photomicrographs of live/dead stained NIH3T3 cells in bilayer hydrogel bars after treatment with Ca^{2+} (50 mM) or EDTA (10 mM) for 10 or 30 min, and subsequent 24 h culture in NIH3T3 GM.

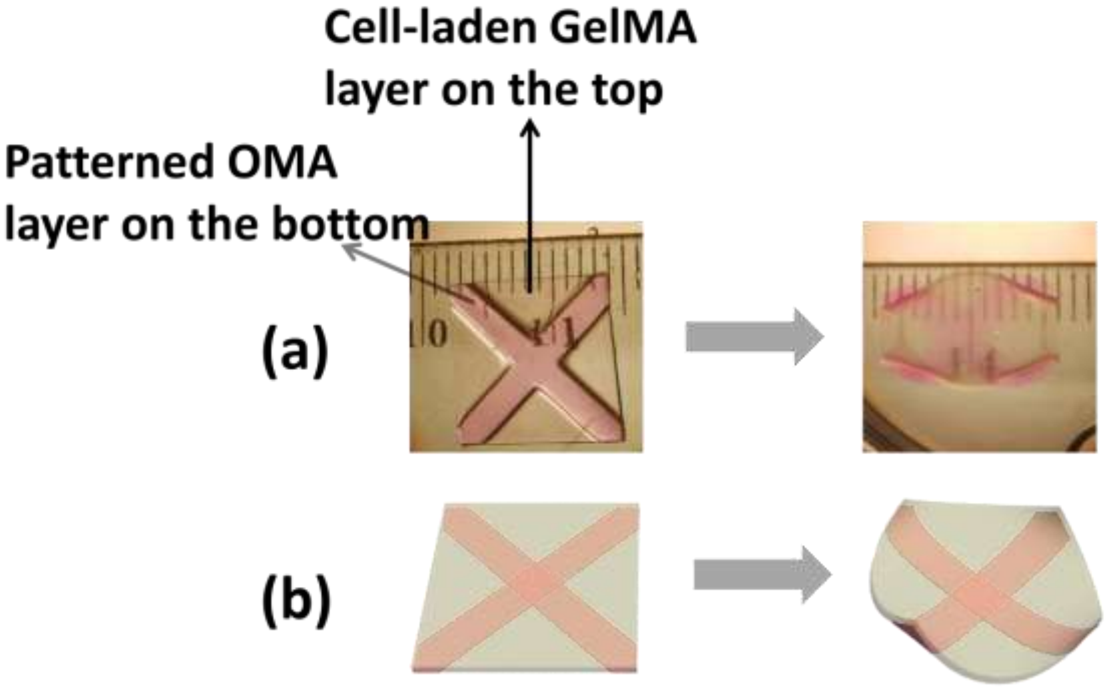

Figure S22. OMA "X" patterned cell-laden GelMA layer folded into a "quasi-four-petal" flower: (a) photomicrographs of a sample and (b) a schematic showing the folding process.

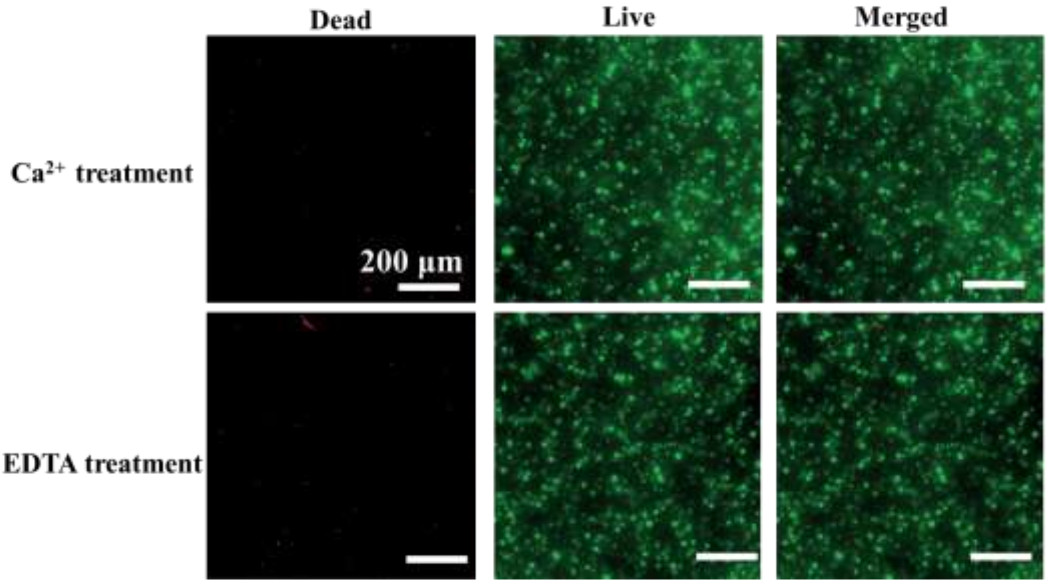

Figure S23. Photomicrographs of live/dead stained NIH3T3 cells in the quasi-four-petal flower after treatment with Ca^{2+} (50 mM, 10 min) or EDTA (5 mM, 20 min).

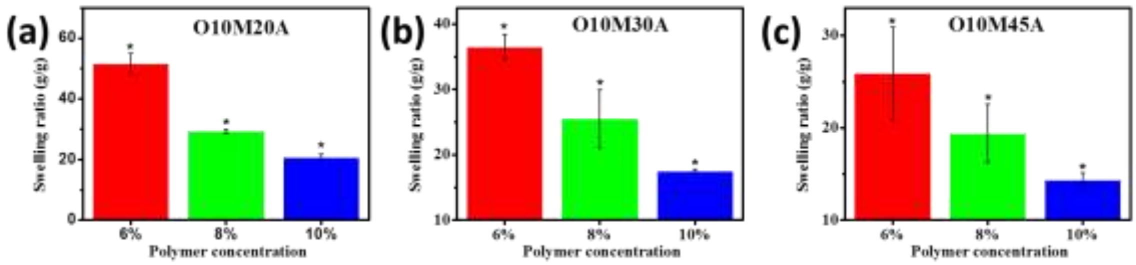

Figure S24. Swelling ratios of (a) O10M20A, (b) O10M30A, and (c) O10M45A hydrogels at different concentrations after 10 hours in DMEM-LG at 4 ºC, *$p < 0.05$ compared to all other groups (one-way ANOVA). Data are presented as mean ± SD, $N = 3$.

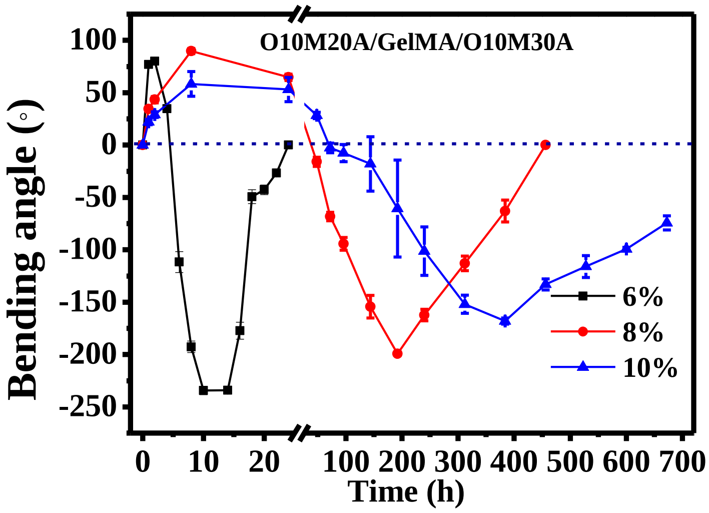

Figure S25. The impact of OMA concentrations (6%, 8%, or 10% in both OMA layers) on the bending angles of the O10M20A/GelMA/O10M30A trilayer hydrogel bar in DMEM-LG. Data are presented as mean ± SD, $N = 3$.

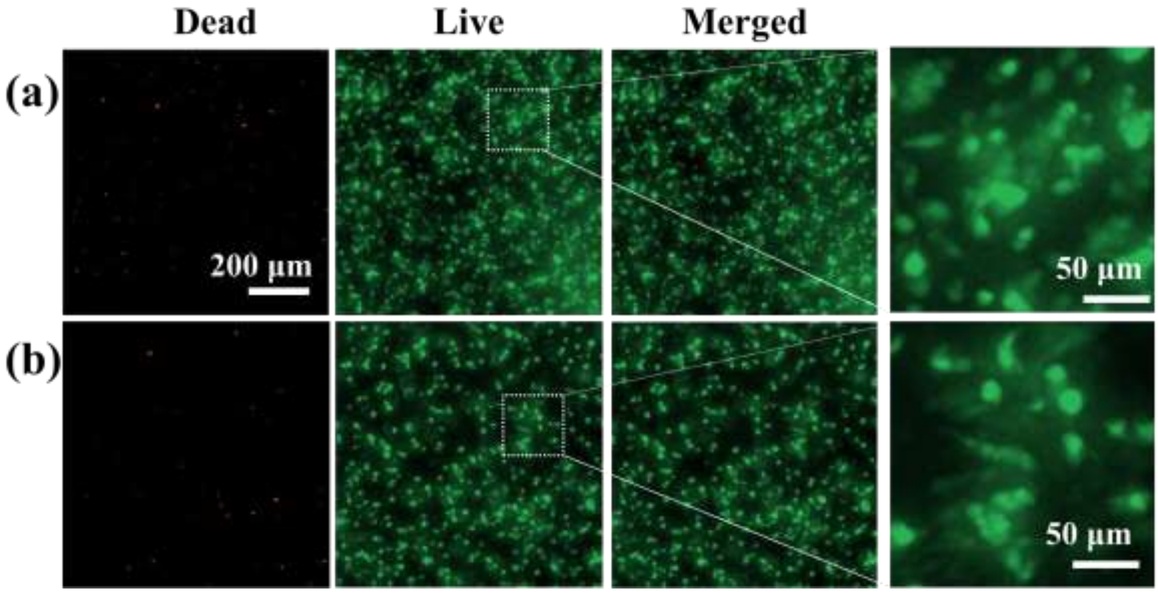

Figure S26. Photomicrograph of live/dead stained hMSCs after (a) 3-week culture in chondrogenic media and (b) 4-week culture in osteogenic media.

References:

[S1] O. Jeon, J.Y. Shin, R. Marks, M. Hopkins, T.H. Kim, H.H. Park, E. Alsberg, *Chem. Mater.* **2017**, *29*, 8425.
[S2] K.Y. Lee, K.H. Bouhadir, D.J. Mooney, *Macromolecules*, **2000**. *33*, 97-101.
[S3] O. Jeon, J.E. Samorezov, E. Alsberg, *Acta biomater.* **2014**, *10*, 47-55.
[S4] C. Ma, W. Lu, X. Yang, J. He, X. Le, L. Wang, J. Zhang, M.J. Serpe, Y. Huang, T. Chen, *Adv. Func. Mater.* **2018**, *28*, 1704568.
[S5] M.K. Nguyen, O. Jeon, M.D. Krebs, D. Schapira, E. Alsberg, *Biomaterials*, **2014**, 35, 6278.
[S6] A. D. Dikina, H. A. Strobel, B. P. Lai, M. W. Rolle, E. Alsberg, *Biomaterials*, **2015**, 52, 452.
[S7] C. A. Schneider, W.S. Rasband, K.W. Eliceiri, 2012, *Nat*, *Methods*, *9*, 671-675.
[S8] A. Kirillova, R. Maxson, G. Stoychev, C.T. Gomillion, L. Ionov, 2017, *Adv. Mater.*, *29*, 1703443.
[S9] O Jeon., Y.B. Lee, H. Jeong, S.J. Lee, D. Wells, E. Alsberg, *Mater. Horiz.* **2018**, *6*, 1625.
